# Semenogelins Armed in Zn(II) and Cu(II): May Bioinorganic Chemistry Help Nature to Cope with *Enterococcus faecalis*?

**DOI:** 10.1021/acs.inorgchem.3c02390

**Published:** 2023-08-15

**Authors:** Dorota Dudek, Adriana Miller, Aleksandra Hecel, Arian Kola, Daniela Valensin, Aleksandra Mikołajczyk, Miquel Barcelo-Oliver, Agnieszka Matera-Witkiewicz, Magdalena Rowińska-Żyrek

**Affiliations:** † Faculty of Chemistry, University of Wrocław, 50-383 Wrocław, Poland; ‡ Department of Biotechnology, Chemistry and Pharmacy, University of Siena, 53100 Siena, Italy; § Screening of Biological Activity Assays and Collection of Biological Material Laboratory, Wroclaw Medical University Biobank, Faculty of Pharmacy, Wrocław Medical University, 50-556 Wroclaw, Poland; ∥ Department of Chemistry, University of Balearic Islands, 07122 Palma de Mallorca, Spain

## Abstract

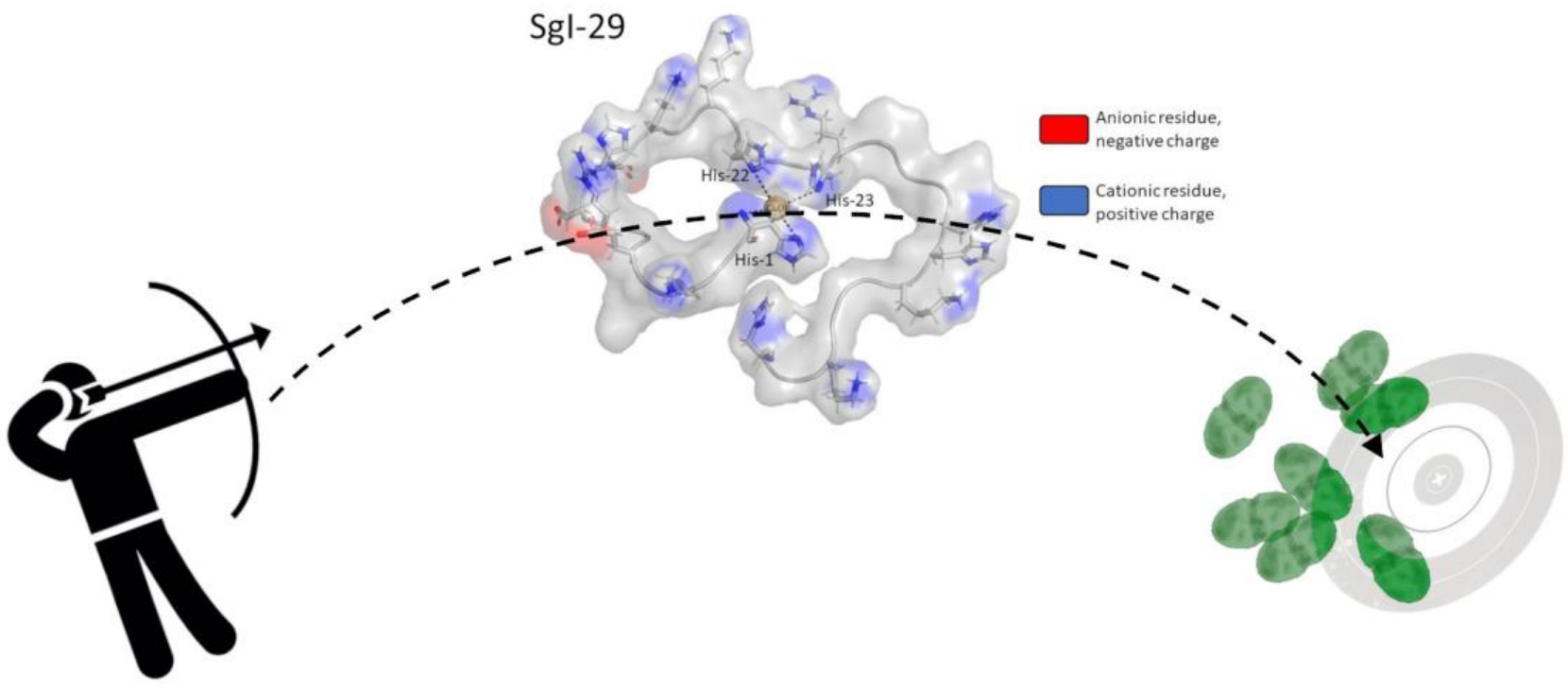

Proteolytic degradation of semenogelins, the most abundant proteins from human semen, results in the formation of 26- and 29-amino acid peptides (SgIIA and SgI-29, respectively), which share a common 15 amino acid fragment (Sg-15). All three ligands are effective Zn(II) and Cu(II) binders; in solution, a variety of differently metalated species exist in equilibrium, with the [NH_2_, 3N_im_] donor set prevailing at physiological pH in the case of both metals. For the first time, the Cu(II)-induced antimicrobial activity of Sg-15 against *Enterococcus faecalis* is shown. In the case of the two native semenogelin fragment metal complexes, the strong local positive charge in the metal-bound HH motif correlates well with their antimicrobial activity. A careful analysis of semenogelins’ metal coordination behavior reveals two facts: (i) The histamine-like Cu(II) binding mode of SgI-29 strongly increases the stability of such a complex below pH 6 (with respect to the non-histamine-like binding of SgIIA), while in the case of the SgI-29 Zn(II)-histamine-like species, the stability enhancement is less pronounced. (ii) The HH sequence is a more tempting site for Cu(II) ions than the HXH one.

## Abstract 2

Antimicrobial fragments of semenogelins from the human semen (peptides: SgIIA, SgI-29, and their common 15 amino acid fragment, Sg-15) bind Zn(II) and Cu(II) ions via a [NH_2_, 3Nim] donor set at physiological pH, and metal binding enhances their antimicrobial activity. In the case of the two native semenogelin fragment metal complexes, the strong local positive charge in the metal-bound HH motif correlates well with their antimicrobial activity.

## 1 Introduction

Semenogelin I (SgI) and semenogelin II (SgII) are dominant proteins of the human semen plasma.^1^ Their sequences share 78% homology, which includes 60 amino acid (AA) conservative motifs, repeated six times in SgI and eight times in SgII (Supplementary Figure S1).^2^

The synthesis and storage of semenogelins occur in the glandular epithelium of the seminal vesicles. They constitute about 60% of the ejaculate volume, which also includes sperm produced in the epididymis (about 5%) and serine proteases produced in the prostate gland (mainly prostate-specific antigen (PSA), about 30%). During ejaculation, all fractions combine to form a gel-like coagulate. An important role in this process is played by Zn^2+^ ions, which concentration in the semen plasma (ca. 2 mM) is about 100 times higher than in blood plasma.

The secretion of the prostate gland consists of PSA and free Zn^2+^, which inhibits the activity of PSA. Zn^2+^ also binds to semenogelins, triggering their conformational change and allowing the formation of a gel coagulate, which is designed to immobilize sperm. At the same time, the concentration of free Zn^2+^ ions decreases, which causes PSA activation. A proteolytic decomposition of semenogelins occurs, resulting in the destruction of the coagulate matrix and allowing of sperm to move freely.^3^

In addition to participating in the process of coagulation and liquefaction of sperm, semenogelins can serve as substrates for transglutaminase and activators of sperm hyaluronidase, which are responsible for the degradation of the envelope around the oocyte, which is important in the fertilization process.^4^ Interestingly, semenogelins have also been found in other organs such as kidneys, trachea, and the retina of the eye; however, their role has not yet been sufficiently clarified.^1^

It is suggested that semenogelins also have an antimicrobial function. In a study by H. Zhao et al.,^5^ four fragments of semenogelin I, formed as a result of proteolytic decay, were identified. One of them, SgI-29, with the amino acid sequence KQEGRDHDKSKGHFHRVVIHHKGGKA, was found to exhibit antibacterial properties against specific isolated *Escherichia coli* (*E. coli*) and *Pseudomonas aeruginosa* (*P. aeruginosa*) clinical strains.^5^ Furthermore, for SgII peptide A, with the sequence KQEGRDHDKSKGHFHMIVIHHKGGQAHHG, antimicrobial activity against *Streptococci*, *E. coli*, *Staphylococcus aureus* (*S. aureus*), and *Enterococcus faecalis* (*E. faecalis*), as well as moderate effects against *P. aeruginosa* was determined.^6^ Interestingly, it is also proposed that the antimicrobial activity of semenogelin fragments is Zn^2+^-dependent–the removal of Zn^2+^ ions from the semen plasma causes almost a complete inhibition of semenogelins’ antimicrobial properties, while Zn^2+^ readdition restores their antibacterial activity.^6^ In contrast, Edstrom et al.^6^ did not clearly confirm the antimicrobial effect of SgI and SgII as it was proposed by Bourgeon et al.^7^ Taken together, this urged us to elucidate the semenogelin Zn^2+^- and Cu^2+^-binding modes and to understand the relationship between the complexes’ coordination, structure, stability, and mode of action, keeping in mind that in general, metal ions may have a dual effect on the activity of antimicrobial peptides (AMPs): (i) AMPs bind them, so that microbes cannot get enough metals essential for their life and virulence (removal of metal ions, nutritional immunity) or (ii) AMPs need the given metal ion to boost their antimicrobial activity via affecting their charge and/or structure.^8−14^

**Figure 1:**
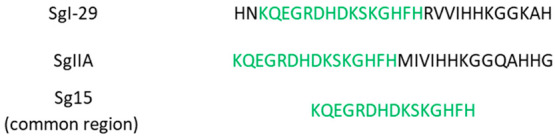
Amino acid sequences of the studied semenogelin fragments. In green, a common fragment is marked with repeating sequences.

In this work, we focus on two semenogelin fragments, SgI-29 and SgIIA, which have been attributed antimicrobial properties in the literature, and one shorter fragment, Sg-15, that has not been studied so far and is a 15 AA fragment of SgI-29 (residues 3–17) and SgIIA (residues 1–15). The amino acid sequences of all three ligands are shown in Figure 1.

Throughout this work, the typical 1:1 metal to peptide ratio is studied; however, it has to be noted that we cannot be certain about the biologically relevant ratio, since the semen Zn(II) and Cu(II) concentrations vary significantly between individuals–differences reaching more than 2 orders of magnitude are described. Supplementary Table S1 presents literature data indicating concentrations of metals in semen samples.

## 3 Experimental Section

### 3.1 Materials

All peptides (KQEGRDHDKSKGHFH–COOH, HNKQEGRDHDKSKGHFHRVVIHHKGGKAH–COOH, KQEGRDHDKSKGHFHMIVIHHKGGQAHHG–COOH) were purchased from KareBay Biochem (USA) (certified purity: 98%) and were used as received. The carbonate-free stock solutions of 0.1 M NaOH were purchased from Sigma-Aldrich and then potentiometrically standardized with the primary standard of potassium hydrogen phthalate (99.9% purity).

### 3.2 Mass Spectrometry

High-resolution mass spectra were obtained on a Bruker Apex Ultra FT-ICR (Bruker Daltonik, Bremen, Germany), equipped with an Apollo II electrospray ionization source with an ion funnel and LCMS-9030 qTOF Shimadzu (Shimadzu, Kyoto, Japan) device, equipped with a standard ESI source and the Nexera X2 system. Bruker Apex Ultra FT-ICR mass spectrometer was operated in positive ion mode. The instrumental parameters were as follows: scan range *m*/*z* 100–2000, dry gas–nitrogen, temperature 473 K, and ion energy 5 eV. The capillary voltage was optimized to the highest S/N ratio, and it was 4200 V. LCMS-9030 qTOF Shimadzu was operated in positive and negative ion modes. The instrumental parameters were as follows: scan range *m*/*z* 100–2000, nebulizing gas nitrogen, nebulizing gas flow 3.0 L/min, drying gas flow 10 L/min, heating gas flow 10 L/min, interface temperature 300^◦^C, desolvation line temperature 400^◦^C, detector voltage 2.02 kV, interface voltage 4.0 kV, collision gas argon, mobile phases (A) H_2_O + 0.1% HCOOH, (B) MeCN + 0.1% HCOOH, mobile phase total flow 0.3 mL/min. The injection volume was optimized depending on the intensity of the signals observed on the mass spectrum within the range of 0.1 to 3 μL. The samples were prepared in a 1:1 methanol–water mixture with a M^2+^/L molar ratio 1:1, [ligand] = 3 × 10^–4^ M, pH 7.4. The samples were infused at a flow rate of 3 μL min^–1^. The instrument was calibrated externally with a Tunemix mixture (Bruker Daltonik, Germany) in quadratic regression mode. Data were processed using Bruker Compass DataAnalysis 4.0 and ACDLabs Spectrus Processor v2021.1.3 programs. The mass accuracy for the calibration was better than 5 ppm, enabling together with the true isotopic pattern (using SigmaFit) an unambiguous confirmation of the elemental composition of the obtained complex.

### 3.3 Potentiometry

Stability constants for proton, Zn^2+^, and Cu^2+^ complexes were calculated from pH-metric titration curves carried out over the pH range 2–11 at *T* = 298 K in a water solution of 4 mM HClO_4_ and 100 mM NaClO_4_, using a total volume of 3 mL. The potentiometric titrations were performed using a Metrohm Titrando 905 titrator and a Mettler Toledo InLab Micro combined pH electrode. The thermostabilized glass-cell was equipped with a magnetic stirring system, a microburet delivery tube and an inlet–outlet tube for argon. Solutions were titrated with 0.1 M carbonate-free NaOH. A standard waiting time of 10–1000s was used between each NaOH addition of 0.003 mL. Each of the systems (peptide, Zn(II) and Cu(II) complexes) was measured three times, and the calculated constants were based on all three measurements. The electrodes were calibrated daily for hydrogen ion concentration by titrating HClO4 with NaOH under the same experimental conditions as above. The purities and the exact concentrations of the ligand solutions were determined by the Gran method.^15^ The ligand concentration was 0.5 mM. The Zn^2+^ and Cu^2+^ to ligand ratio was 1:1. The standard potential and the slope of the electrode couple were computed by means of Glee program.^16^ The HYPERQUAD 2006^17^ program was used for the stability constant calculations. The standard deviations were computed by HYPERQUAD 2006 and refer to random errors only. The constants for the hydrolytic Zn^2+^ and Cu^2+^ species were used in these calculations. The speciation and competition diagrams were computed with the HYSS program.^18^ In order to ensure the reversibility of the titrations, all samples were acidified with HClO_4_ (with the exact amount of moles of NaOH that were used during the first titration) and retitrated, in order for the titration curve to be compared to the original one.

### 3.4 Spectroscopic Measurements

The absorption spectra were recorded on Varian Cary300 Bio spectrophotometer, in the range 200–800 nm, using a quartz cuvette with an optical path of 1 cm. Circular dichroism (CD) spectra were registered on a Jasco J-1500 CD spectrometer in the 200–800 nm range, using a quartz cuvette with an optical path of 1 cm or with a cuvette with an optical path of 0.01 cm in the wavelength range 180–300 nm. The solutions were prepared in a water solution of 4 mM HClO4 at an ionic strength of *I* = 100 mM (NaClO_4_). The concentrations of solutions used for spectroscopic studies were similar to those in the potentiometric experiments; Cu^2+^: ligand ratio was also 1:1. The UV–vis and CD spectroscopic parameters were calculated from the spectra obtained at the pH values corresponding to the maximum concentration of each particular species, based on distribution diagrams.

NMR experiments were carried out at 278 and 298 K on a 600 MHz Bruker Advance spectrometer. NMR spectra were processed with TopSpin 3.6 software and analyzed with the program Sparky.^19^ Suppression of residual water signal was achieved by excitation sculpting,^20^ using a selective 2 ms long square pulse on water. Proton resonance assignment was achieved by 2D NMR analysis, ^1^H–^1^H TOCSY and NOESY. The peptides were dissolved in water with 10% of D_2_O and the pH was adjusted by adding small volumes of HCl or NaOH. The final peptide concentration was 1 mM. The desired concentrations of Zn^2+^ and Cu^2+^ ions were obtained by using stock solutions of Zn(NO_3_)_2_ and Cu(NO_3_)_2_.

### 3.5 Antimicrobial Activity Assay of Free Peptide and Peptide–Metal Ion Complex

Seven reference strains from ATCC collection (*P. aeruginosa* 27853, *E. coli* 25922, *S. aureus* 43300, *E. faecalis* 29212, *Klebsiella pneumoniae* 700603, *Acinetobacter baumannii* 19606, and *Candida albicans* 10231) were used for antimicrobial activity assay. The antimicrobial effect of analyzed peptides/complexes was performed according to the standard protocol using two protocols: microdilution method with spectrophotometric measurement (λ = 580 nm) according to the ISO standard 20776-1:2019,^21^ ISO standard 16256:2012,^22^ and modified Richard’s method.^23−25^ After 24 h/310 K (for bacteria) or 24 h/298 K (for fungus) in tryptone soy broth medium (TSB) incubation, the density of bacterial and fungal suspension was measured, a proper dilution was prepared (0.005MF, 5 × 10^5^ CFU/mL for bacteria and 0.025 MF, 0.5–2.5 × 10^5^ CFU/mL for fungus).^23,26^ Afterward, a 96-well microplate was prepared where the concentration range 256 μg/mL–0.5 μg/mL of ligand/complex solution was examined. The final bacterial and fungal inocula were 2.5 × 10^4^ CFU/well for bacteria and 2.5 × 10^3^–1.25 × 10^4^ CFU/well for fungus.

Positive (TSB + strain) and negative (TSB) controls were also included in the test. Microplates were incubated at 37 ± 1 °C (bacteria) and 25 ± 1 °C (fungus) for 24 h on the shaker. After this, spectrophotometric measurements were done. Minimal inhibitory concentration (MIC50) was defined as the lowest concentration of an antimicrobial agent that decreased the measured microbial growth to 50% as referred to as positive control. To validate the assay, antibacterial/antifungal agents compatible with each strain (*E. faecalis* 29212: levofloxacin 4 μg/mL, *P. aeruginosa* 27853 and *S. aureus* 43300: levofloxacin 1 μg/mL, *A. baumannii* 19606: levofloxacin 0.5 μg/mL, *E. coli* 25922 and *K. pneumoniae* 700603: gentamicin 4 μg/mL, *C. albicans* 10231: amphotericin B 1 μg/mL) were tested against each strain.

After, 50 μL aliquots of 1% (m/v) 2,3,5-triphenyltetrazolium chloride (TTC) solution was added in each well. TTC is converted to red formazan crystals in microbial live cells. MBC/MFC can be observed as the lowest concentration that did not show microbial growth by visual analysis after 24 h of incubation with TTC (did not change the color to pink). Thanks to both methods, potential MIC50, MBC, or MFC can be determined.

## 4 Results and Discussion

### 4.1 Sg-15 (KQEGRDHDKSKGHFH)

Sg-15 contains 11 groups that are involved in the acid–base reaction. Deprotonating groups correspond to the C-terminal carboxylic group, two aspartic acid side chain carboxylates, glutamic acid side chain carboxylate, three histidine imidazoles, N-terminal amine and three lysine side chains with p*K*_*a*_ values 2.34, 3.16, 3.58, 4.36, 6.06, 6.46, 7.1, 7.58, 9.66, 10.53, and 10.66, respectively (Table 1). Sg-15 does not adopt any predefined structural preference in solution, as indicated by the absence of nontrivial NOEs correlations in the ^1^H–^1^H NOESY spectra recorded at both acidic and physiological pH. Finally, NMR spectra recorded by lowering temperature to 278 K confirmed the lack of specific structural rearrangements adopted by Sg-15.

**Table 1:** Potentiometric Data for Proton, Zn^2+^, and Cu^2+^ Complexes with Sg-15 (KQEGRDHDKSKGHFH), SgI-29 (HNKQEGRDHDKSKGHFHRVVIHHKGGKAH), and SgIIA (KQEGRDHDKSKGHFHMIVIHHKGGQAHHG)^a^

	Sg-15 KQEGRDHDKSKGHFH		SgI-29 HNKQEGRDHDKSKGHFHRVVIHHKGGKAH		SgIIA KQEGRDHDKSKGHFHMIVIHHKGGQAHHG	
species	log β	log *K*	log β	log *K*	log β	log *K*
HL	10.66 (3)	10.66				
H_2_L	21.19 (1)	10.53	22.24 (4)		22.84 (4)	
H_3_L	30.85 (2)	9.66	32.71 (2)	10.47	33.18 (3)	10.34
H_4_L	38.43 (2)	7.58	43.09 (3)	10.38	43.39 (4)	10.21
H_5_L	45.53 (2)	7.10	52.98 (2)	9.89	52.86 (3)	9.47
H_6_L	51.99 (2)	6.46	62.43 (3)	9.45	60.41 (3)	7.55
H_7_L	58.05 (2)	6.06	69.82 (3)	7.39	67.51 (4)	7.10
H_8_L	62.41 (2)	4.36	76.75 (3)	6.93	74.10 (3)	6.59
H_9_L	65.99 (2)	3.58	83.24 (3)	6.49	80.51 (4)	6.41
H_10_L	69.15 (3)	3.16	89.48 (3)	6.24	86.47 (3)	5.96
H_11_L	71.49 (4)	2.34	95.30 (3)	5.82	92.22 (3)	5.75
H_12_L			100.82 (3)	5.52	97.42 (3)	5.20
H_13_L			105.71 (3)	4.89	101.68 (3)	4.26
H_14_L			109.45 (3)	3.74	104.68 (3)	3.00
H_15_L			112.67 (3)	3.22	107.45 (3)	2.77
H_16_L			114.87 (3)	2.20		
H_17_L			117.05 (4)	2.18		
Zn^2+^ Complexes						
ZnH_10_L			93.34 (3)			
ZnH_9_L			87.85 (3)	5.49		
ZnH_8_L			82.14 (2)	5.71	78.58 (2)	
ZnH_7_L			75.94 (1)	6.20	72.91 (1)	5.67
ZnH_6_L			69.11 (1)	6.83	66.44 (1)	6.47
ZnH_5_L			61.34 (1)	7.77	59.44 (1)	7.00
ZnH_4_L	44.08 (1)		52.80 (1)	8.54	51.44 (1)	8.00
ZnH_3_L	37.20 (2)	6.88	43.49 (2)	9.31	42.46 (2)	8.98
ZnH_2_L	29.06 (3)	8.14	33.75 (3)	9.74	32.65 (2)	9.81
ZnHL	20.17 (3)	8.89	23.68 (4)	10.07	22.60 (1)	10.05
ZnL	10.65 (3)	9.52	13.09 (5)	10.59		
ZnH_–1_L	0.25 (4)	10.40	2.49 (3)	10.60		
ZnH_–2_L	–10.34 (3)	10.59				
Cu^2+^ complexes						
CuH_12_L			105.41 (3)			
CuH_11_L			101.77 (1)	3.64	97.29 (1)	
CuH_10_L			97.32 (2)	4.45		
CuH_9_L			92.61 (1)	4.71	88.33 (1)	
CuH_8_L			87.15 (1)	5.46	83.15 (1)	5.18
CuH_7_L			80.96 (1)	6.19	77.84 (1)	5.31
CuH_6_L			74.3 (1)	6.66	71.95 (1)	5.89
CuH_5_L	52.48 (1)		66.39 (2)	7.91	65.56 (1)	6.39
CuH_4_L	47.09 (1)	5.39	57.76 (3)	8.63	58.72 (1)	6.84
CuH_3_L	40.27 (2)	6.82	48.93 (2)	8.83	50.84 (1)	7.88
CuH_2_L	32.95 (2)	7.32	39.22 (3)	9.71	41.99 (1)	8.85
CuHL	24.54 (3)	8.41	29.17 (3)	10.05	32.51 (1)	9.48
CuL	15.27 (2)	9.27	19.1 (2)	10.07	22.43 (1)	10.08
CuH_–1_L					11.79 (1)	10.64
CuH_–2_L	–5.44 (2)		–2.29 (1)		1.15 (1)	10.64

a Titrations were carried out over the pH range 2–11 at 298 K in aqueous solution with 4 mM HClO_4_ and 0.1 M NaClO_4_. The peptide concentration was 0.5 mM and the metal-to-peptide ratio was 1:1. HYPERQUAD 2006 was used to determine the stability constants. Standard deviations are shown in brackets. N-t refers to the N-terminal amine group.

#### 4.1.1 Zinc(II) Complexes

ESI-MS confirmed the stoichiometry of the Zn^2+^ semenogelin complexes. Supplementary Figure S2A shows the spectra for the Zn^2+^-Sg-15 complexes.

Comparison of the measured and simulated isotopic patterns confirms the presence of the mentioned Zn^2+^ complexes only in a mononuclear form (with a ligand/metal ratio of 1:1; Supplementary Figure S2B).

**Figure 2:**
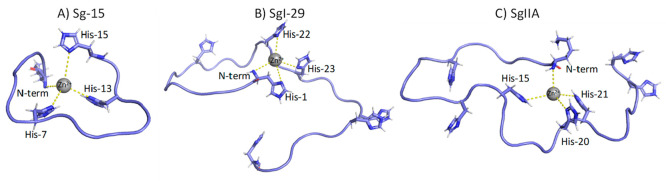
Predicted structural models of Zn^2+^ binding sites for: (A) Sg-15 – N-terminal amine group and 3 imidazoles from His7, His13, and His15; (B) SgI-29 – N-terminal amine group and 3 imidazoles from His1, His22, and His23, and (C) SgIIA – N-terminal amine group and 3 imidazoles from His15, His20, and His21, at pH 7.4.

The Zn(II) complex formed in acidic conditions is ZnH_4_L, where most probably all four acidic groups are already deprotonated and all three imidazoles (His-7, His-13 and His-15) are involved in coordination (Table 1, Supplementary Figure S3A). It is worth noting that Zn(II) (and also Cu(II)) ions are intermediate Pearson’s acids with mutual affinity with N-heterocyclic atoms, which are intermediate Pearson’s bases. Also highlighting that among the mammalian amino acid side chains, the histidine one is the only one that efficiently provides such N-heterocyclic donors. With the increase of pH, ZnH_3_L species are observed with a p*K*_*a*_ of 6.88, involving the N-terminal amine in the coordination sphere. At this point, the coordination mode {3N_im_, NH_2_} shown in Figure 2A, is in good agreement with the NMR findings at pH 7.4 (*vide infra*).

The comparison of ^1^H NMR spectra recorded in absence and in the presence of Zn^2+^ ions allowed to identify the metal binding domains of Sg-15. NMR analysis was initially performed by looking at the variations of chemical shifts and signal line broadenings induced by different metal/peptide ratios (0.125, 0.250, 0.375, 0.500, 0.625, 0.750, and 0.875) as shown in Supplementary Figure S4. At 278 K, the broadest resonances are the backbone amide (H_N_) and His protons, which become barely visible just after the first addition. As expected, increasing the temperature to 298 K results in less signal line-broadening allowing the detection of selective proton chemical shift. On the other hand, H_N_ signals almost vanished at room temperature because of the water–amide proton exchange occurring in both free and metal bound Sg-15 forms.

**Figure 3:**
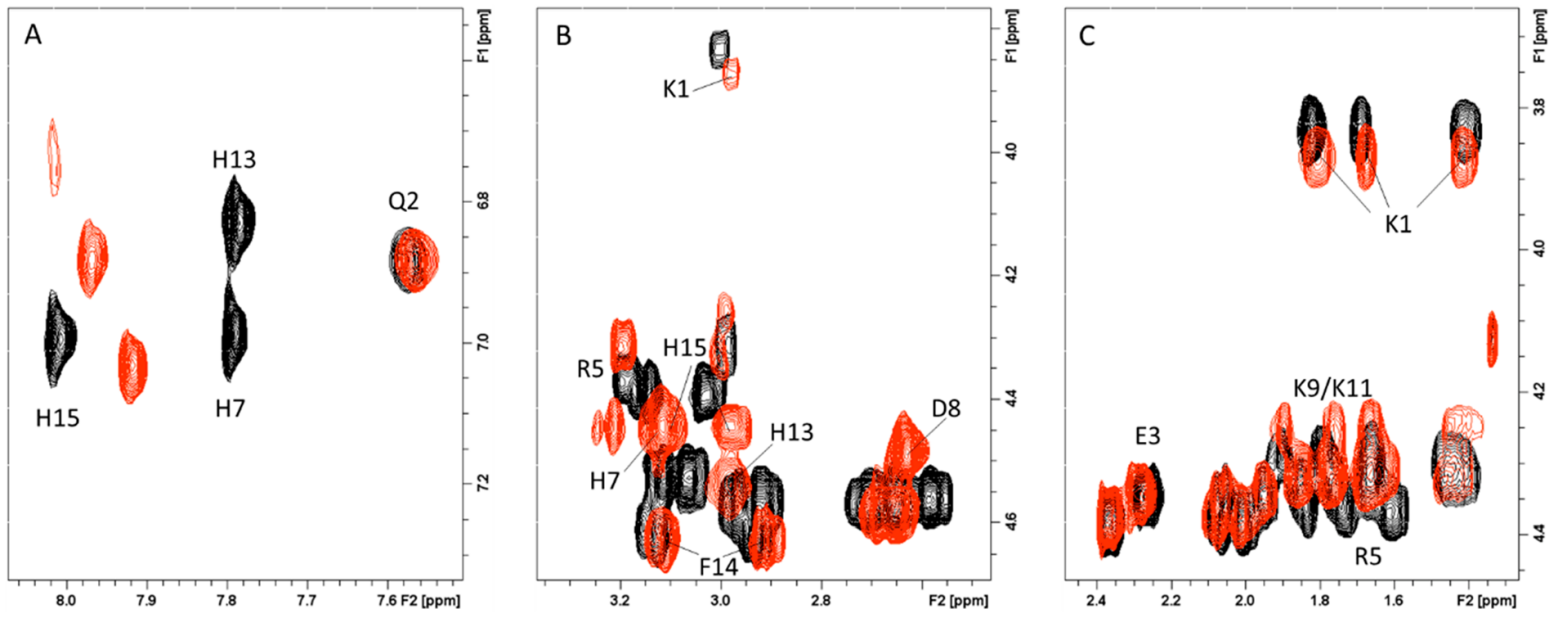
Superimposition of selected regions of ^1^H–^1^H TOCSY spectra of Sg-15, 1 mM in absence (black) and in the presence of 0.875 Zn^2+^ equiv (red), *T* = 298 K, pH 7.4. (A) Aromatic region; (B) and (C) aliphatic regions.

The analysis of 2D ^1^H–^1^H TOCSY NMR spectra recorded before and after the addition of about one zinc(II) equivalent revealed all His as the largest affected residues, as shown by the shifts exhibited by H_δ_–H_ε_ and H_α_–H_β_ correlations (Figure 3A,B). Besides His imidazole nitrogen atoms, variations on Lys-1 H_α_ point out the involvement of the N-terminal amine group in metal binding (Figure 3B,C). The effects observed on Arg-5, Asp-8, Lys-9/11 (signals are overlapped in the free form), and Phe-14 (Figure 3B,C) support the {3N_im_, NH_2_} coordination mode, all of them being in close proximity to residues participating in Zn^2+^ binding. Moreover, the small effects observed on Asp Hβ and Glu Hγ might be explained with the possible involvement of Asp/Glu carboxylic groups in stabilizing the metal binding site, initiated at acidic pH values.

Finally, the 3D structure of the Zn^2+^-Sg-15 complex was investigated by ^1^H–^1^H NOESY spectroscopy. Unfortunately, as expected, the very low intensity of amide proton resonances avoids the detection of well resolved NOEs correlations, preventing the determination of the structural features associated with Zn^2+^-Sg-15 associations.

Loss of one/two protons of ZnH_3_L species leads to the formation of ZnH_2_L and ZnHL species with p*K*_*a*_ values of 8.14 and 8.89, which can be ascribed to the deprotonation of two water molecules bound to the central zinc(II) atom. The remaining three species are related to deprotonation of Lys-1, Lys-9, and Lys-11 side chain groups with p*K*_*a*_ values of 9.52, 10.4, and 10.59, respectively, and have no impact on the complex coordination mode.

#### 4.1.2 Copper(II) Complexes

ESI-MS confirmed that Cu^2+^-Sg-15 complexes exist only in a mononuclear form (with a ligand/metal ratio of 1:1; Supplementary Figure S2C,D).

**Figure 4:**
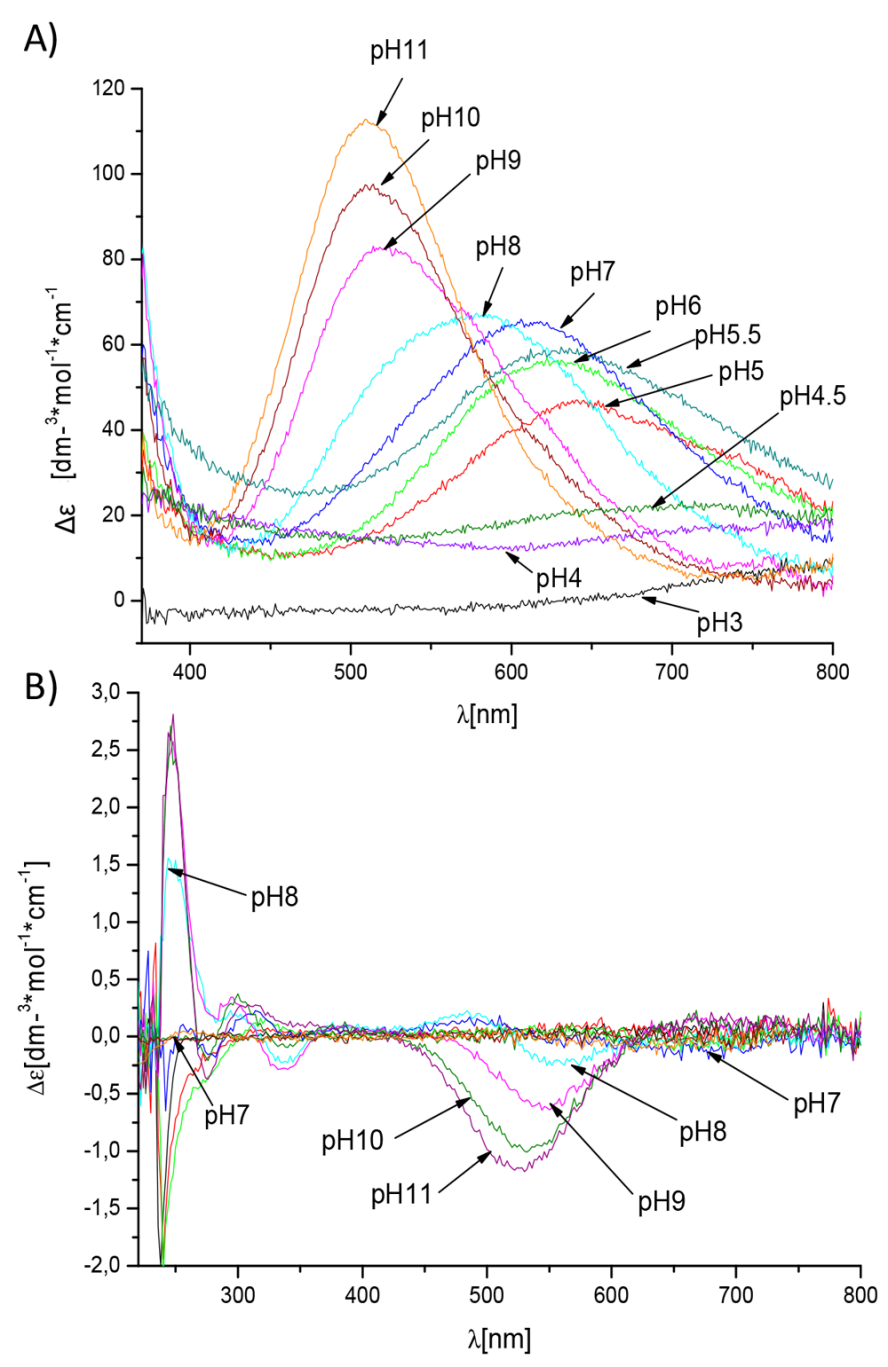
UV–vis (A) and CD (B) spectra of Cu^2+^ complexes with Sg-15 in the range 200–800 nm. M/L ratio = 0.9:1.

Copper(II) starts to interact with Sg-15 around pH 4, and the complexes observed in acidic conditions are CuH_5_L (Table 1, Supplementary Figure S3B). At this point, acidic residues are already deprotonated, and most probably, Cu^2+^ is anchored by two histidine imidazoles. An increasing UV–vis band at around 640 nm confirms the 2N coordination at this pH (Figure 4A, Supplementary Table S2).^27^ The next complex form, CuH_4_L, dominates at around pH 6. Comparison between p*K*a values of the free ligand and the complexed one (7.1 and 5.39, respectively) strongly suggests that a third histidine imidazole is also involved in Cu^2+^ binding. Loss of one proton results in the CuH_3_L complex form, with a maximum at pH 7; here, the N-terminal amine is involved in the coordination sphere, resulting in a {3N_im_, NH_2_} type of coordination.

**Figure 5:**
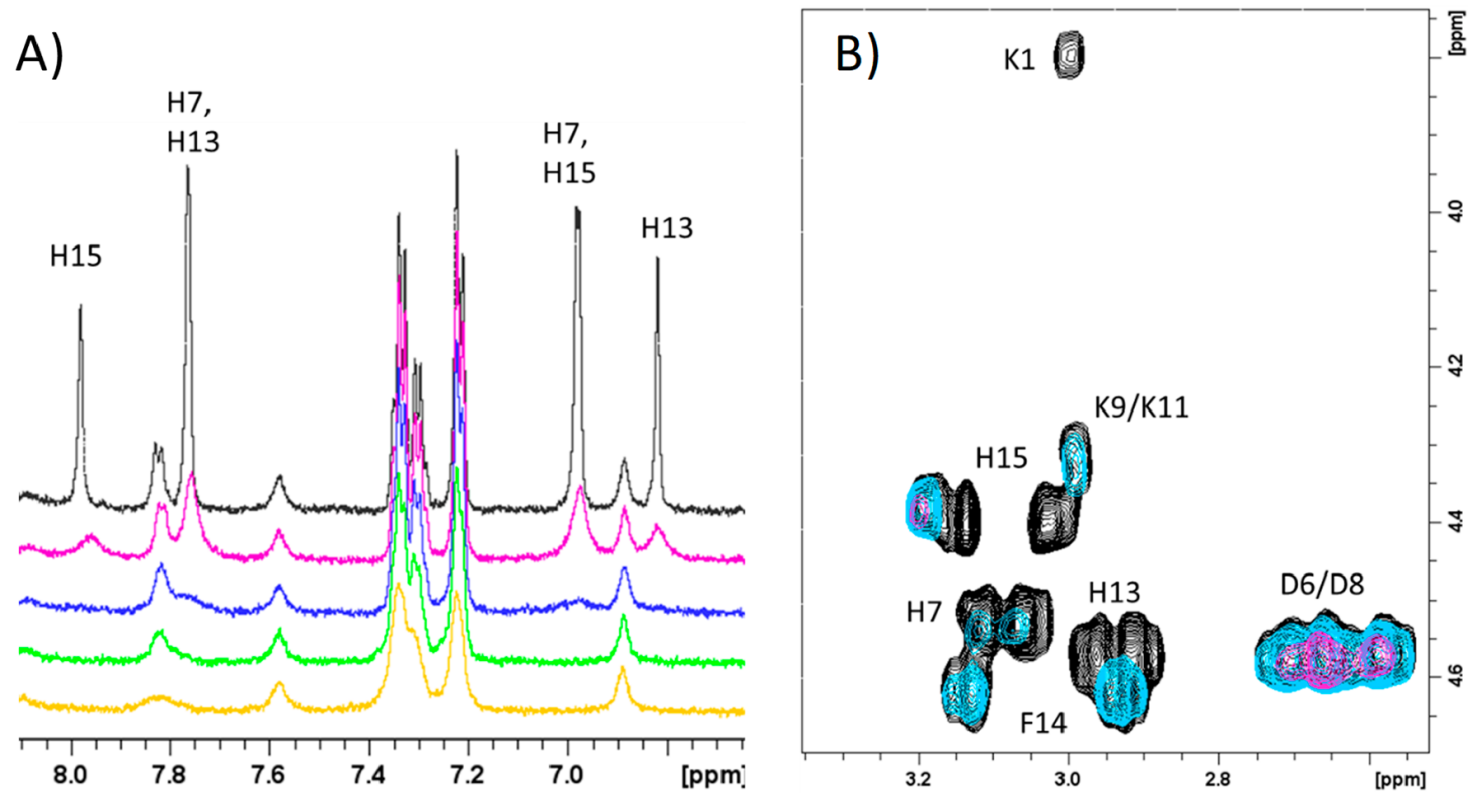
Superimposition of (A) Aromatic region of ^1^H 1D spectra of Sg-15, 1 mM in absence (black) and in the presence of 0.02 (magenta), 0.05 (blue), 0.1 (green), and 0.2 (yellow) Cu^2+^ equiv. (B) Aliphatic region of ^1^H–^1^H TOCSY spectra of Sg-15, 1 mM in absence (black) and in the presence of 0.05 (light blue), 0.2 (magenta) Cu^2+^ equiv. *T* = 298 K, pH 7.4.

**Figure 6:**
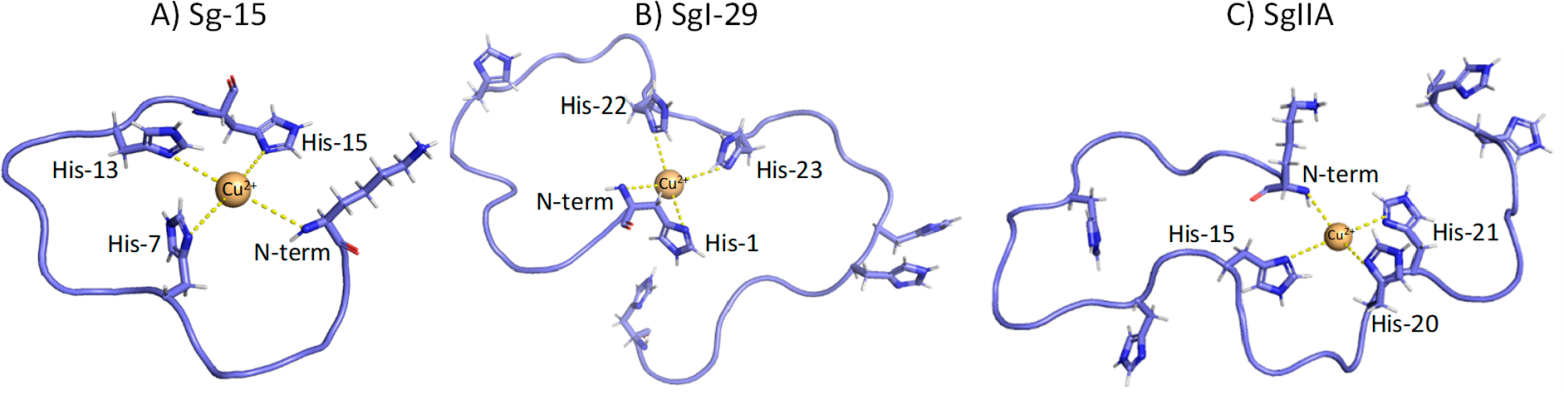
Predicted structural models of Cu^2+^ binding sites for (A) Sg-15 – N-terminal amine group, and 3 imidazoles from His7, His13, and His15; (B) SgI-29 – N-terminal amine group and 3 imidazoles from His1, His22, and His23; and (C) SgIIA – N-terminal amine group and 3 imidazoles from His15, His20, and His21, at pH 7.4.

Copper binding mode in the CuH_3_L species was further investigated by NMR spectroscopy allowing us to identify the residues involved in the metal coordination sphere. Substechiometric copper addition generally leads to severe line broadening of NMR nuclei close to the paramagnetic ion because of the nuclear–electron dipolar couplings.^28−31^ The larger the proximity to metal center, the broader the NMR signal. The identification of the residues interacting with copper is usually obtained by performing titration experiments by gradually increasing Cu^2+^ concentrations until to the complete disappearance of selected NMR resonances.^32,33^Figure 5A indicates that all three His imidazole protons of Sg-15 are broadened upon Cu^2+^ addition at pH 7.4, until they completely disappear in the presence of metal equivalents larger than 0.1. Successive analysis of the aliphatic region of the 2D ^1^H–^1^H TOCSY maps revealed that, besides all the three His, the N-terminal Lys-1 correlation totally disappeared because of the paramagnetic ion effects (Figure 5B). Finally, increasing copper(II) concentrations lead to the broadening of the Phe-14 signal as well, in agreement with its the proximity to His-13 and His-15 residues involved in coordination. The slight broadening on Lys-9 and/or Lys-11 is probably due to copper induced conformational changes in Sg-15 structure. All these data strongly point out the Lys-1 N-terminal amine group and His-7, His-13, and His-15 imidazole nitrogen as the donor atoms participating in the metal binding domain (Figure 6A).

The maximum of the next complex form, CuH_2_L, occurs at pH 8, at which increasing bands in CD spectra near 560 nm (Figure 4B) and in UV–vis band near 575 nm (Figure 4A) are also observed, which indicate a transition between two different coordination modes.^34^ Above this point, an amide nitrogen enters the Cu^2+^ coordination sphere. With the increase of pH, another amide begins to take part in copper(II) coordination (CuHL species), resulting in a {2Nim, 2N^–^} coordination mode. Most probably, Cu^2+^ is anchored to His-15 imidazole, and the coordination proceeds toward the N-terminus, including also amide nitrogens of His-15 and Phe-14 and imidazole nitrogen from His-13. The remaining 3 deprotonations, which result in CuL and CuH_–2_L complex forms, are the result of three lysine side chain deprotonations and have no further impact on the Cu^2+^ coordination mode.

#### 4.2 SgI-29 (HNKQEGRDHDKSKGHFHRVVIHHKGGKAH)

In the case of SgI-29, 16 out of 17 possible formation constants were determined (all except the most basic one, which belongs to one of the Lys side chains and was beyond the working range of the electrode). The first four acidic p*K*_*a*_ values (2.18, 2.2, 3.22, and 3.74) come from deprotonation of the C-terminal carboxylic group, two aspartic acid and one glutamic acid side chains, respectively (Table 1). The following seven p*K*_*a*_ values (4.89, 5.52, 5.82, 6.24, 6.49, 6.93, and 7.39) correspond to the deprotonation of seven histidine imidazoles. Next p*K*_*a*_ value (9.45) comes from the deprotonation of the N-terminal amine group. It seems relatively high, but taking into consideration the high number of histidine residues present in the sequence, the fact that the N-terminal residue is a histidine and the literature data on similar sequences, the value appears to be adequate. The remaining basic p*K*_*a*_ values (9.89, 10.38, and 10.47) come from the deprotonation of lysine residues.

Similar to Sg-15, the NMR analysis of SgI-29 did not reveal the presence of any specific structural rearrangement of the peptide in aqueous solution. All of the spectra recorded at acidic and physiological pH are consistent with a flexible and disordered peptide.

#### 4.2.1 Zinc(II) Complexes

In the ESI-MS spectra we can observe only mononuclear forms of Zn^2+^ complexes with SgI-29 (ligand/metal ratio of 1:1; Supplementary Figure S5A,B).

The first complex form for zinc(II) observed at acidic pH is ZnH_10_L, which starts to occur above pH 4 (Table 1, Supplementary Figure S6A). At this point, seven residues are already deprotonated, and most probably, the zinc(II) coordination sphere includes two histidine imidazoles, while carboxylates of Glu, 2 Asp, C-terminus, and one His imidazole are not involved. Release of two more protons leads to the ZnH_9_L and ZnH_8_L species formation with maxima at pH 5.75 and 6, in which another His imidazole group and N-terminal amine group are being involved into coordination sphere, resulting in an {NH_2_, 3N_im_} coordination mode. Comparison between p*K*_*a*_ values of the free and complexed forms of the next three species (6.49 and 6.20 (ZnH_7_L); 6.93 and 6.83 (ZnH_6_L); and 7.39 and 7.77 (ZnH_5_L)) allows us to conclude that all three of them are not involved in coordination and correlate to the deprotonation of the remaining histidines.

**Figure 7:**
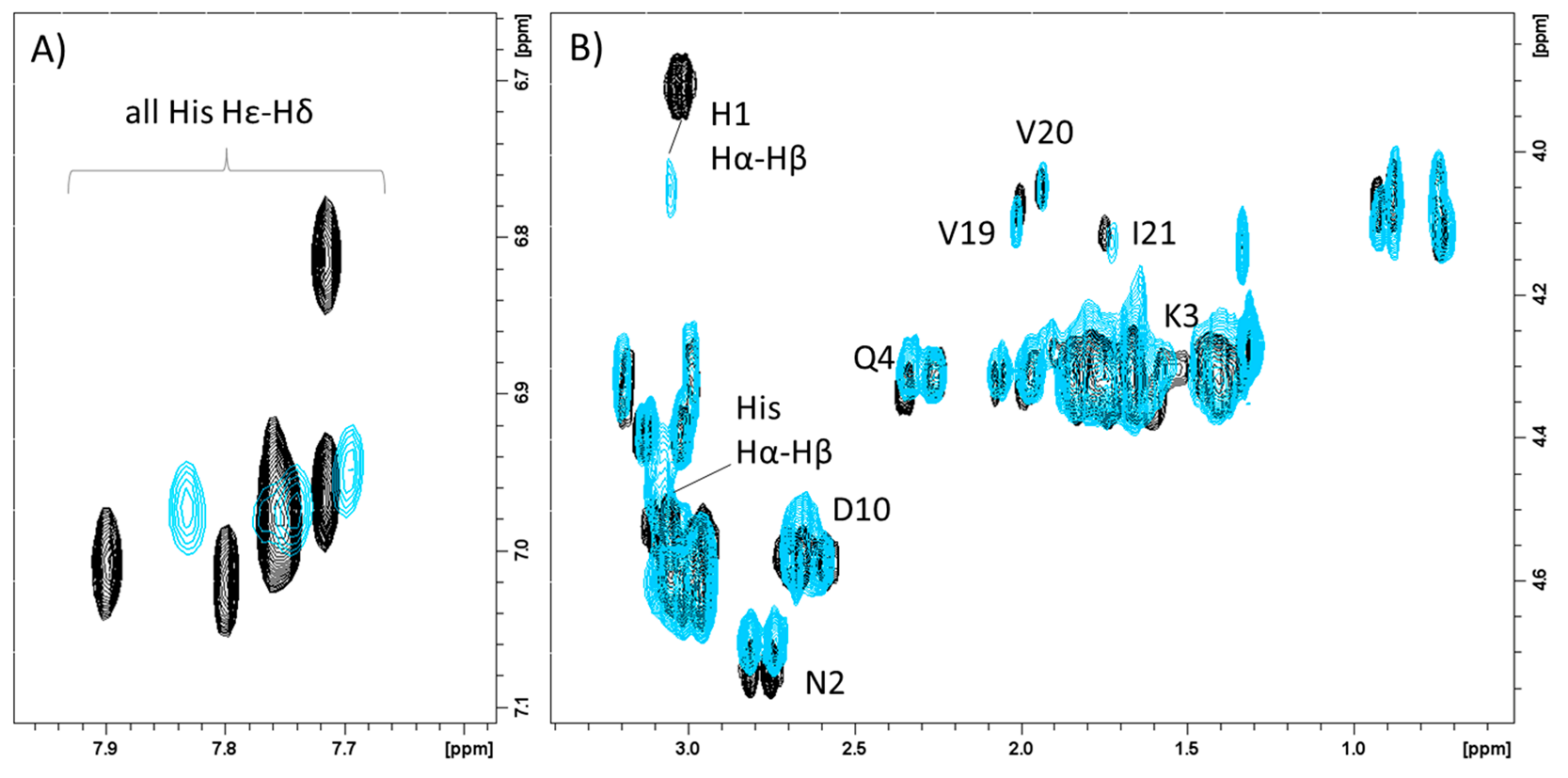
Superimposition of aromatic (A) and aliphatic (B) regions of ^1^H–^1^H TOCSY spectra of SgI-29, 1 mM in absence (black) and in the presence of 0.9 (light blue) equiv of Zn^2+^. *T* = 298 K, pH 7.4.

The structural characterization of the Zn^2+^- SgI-29 complexes was further investigated by monitoring the metal induced variations on NMR spectra. ^1^H–^1^H TOCSY NMR spectra recorded at pH 7.4 (Figure 7A,B), when all of the mentioned species are already formed, reveal that the signals which are most affected by zinc(II) addition belong to the N-terminal His-1, 4 out of 7 histidine imidazole groups, Asn-2, Gln-3, Asp-10, Val-19, Val-20, and Ile-21. On the other hand, the least affected signals, which, at the same time, are in close proximity to histidines or are able to bind zinc(II) itself, belong to Glu-5, Phe-16, and Ala-28. These findings allowed us to exclude His-15, His-17, and His-29 from the metal coordination sphere. Taken together, the most probable donor atoms set for this system at physiological pH are the His-1 N-terminal amine group and imidazole (a so-called histamine-like coordination mode) and two more imidazole nitrogen atoms, presumably His-22 and His-23 (shown in the model reported in Figure 2B), although other combinations including His-9 may also be possible. With increasing pH, loss of the remaining 6 protons leads to the formation of ZnH_4_L, ZnH_3_L, ZnH_2_L, ZnHL, ZnL, and ZnH_–1_L with p*K*_*a*_ values of 8.54, 9.31, 9.74, 10.07, 10.59, and 10.60, respectively, and is related to the deprotonation of one water molecule and five lysine side chain groups, which has no impact on coordination mode.

#### 4.2.2 Copper(II) Complexes

Results from ESI-MS show that Cu^2+^-SgI-29 complexes exist only in a mononuclear form (with a ligand/metal ratio of 1:1; Supplementary Figure S5C,D).

**Figure 8:**
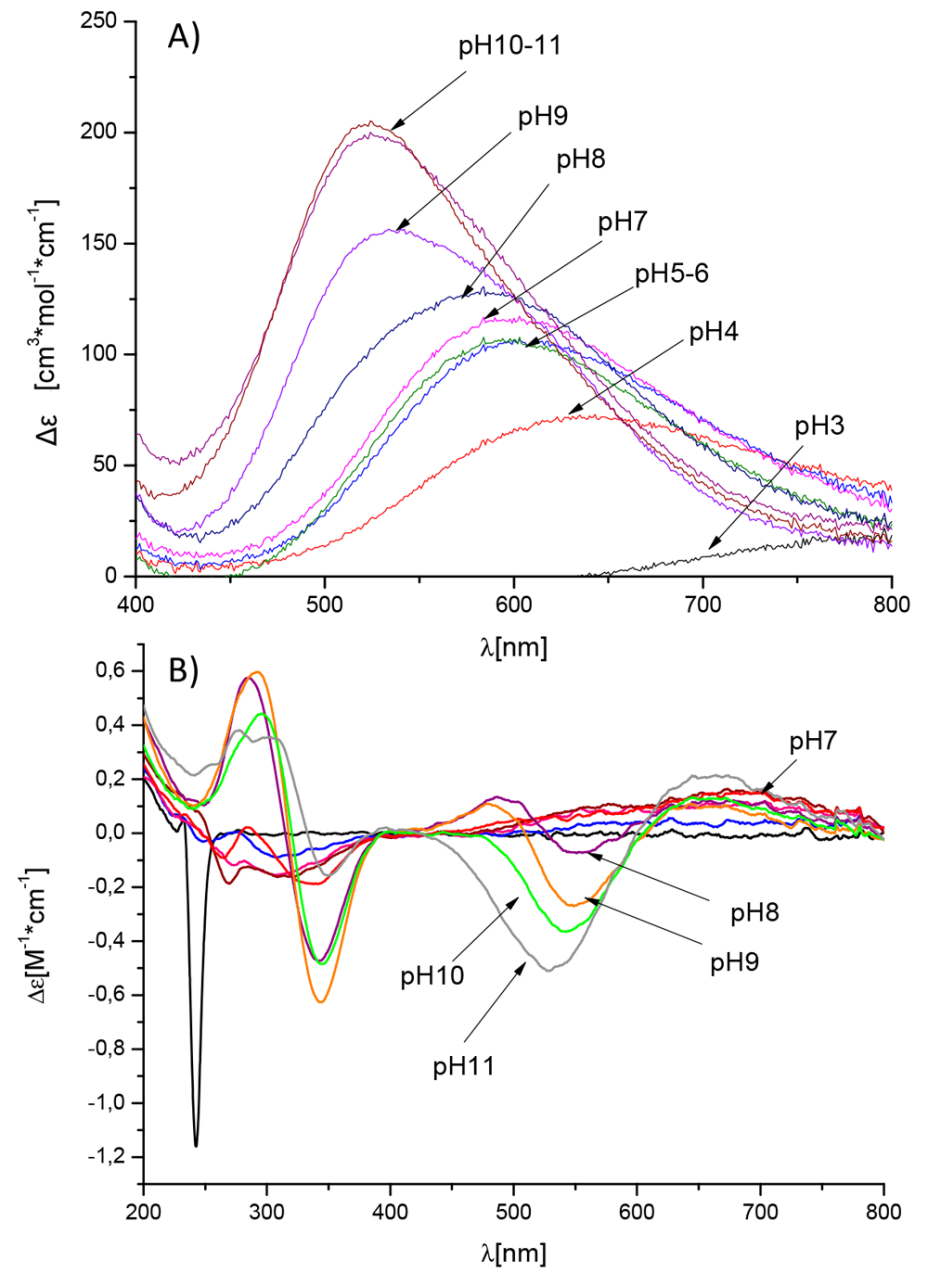
UV–vis (A) and CD (B) spectra of Cu^2+^ complexes with SgI-29 in the range 200–800 nm. M/L ratio = 0.9:1.

The first detected Cu^2+^ complex, CuH_12_L, starts to form at very acidic pH and has a maximum at pH 3.7 (Supplementary Figure S6B). At this point, deprotonation of the C-terminus, three acidic residues (Glu-5, Asp-8, Asp-10) and one of the histidine imidazoles has already taken place (Table 1). Deprotonation of His side chain at such low pH is possible only if it is bound to metal ion. In the next form, CuH_11_L, another imidazole, is involved in copper(II) coordination. The maximum of this form is around pH 4, in which UV–vis spectra confirms the 2N coordination with the increasing band near 635 nm (Figure 8A, Supplementary Table S2).^27^

**Figure 9:**
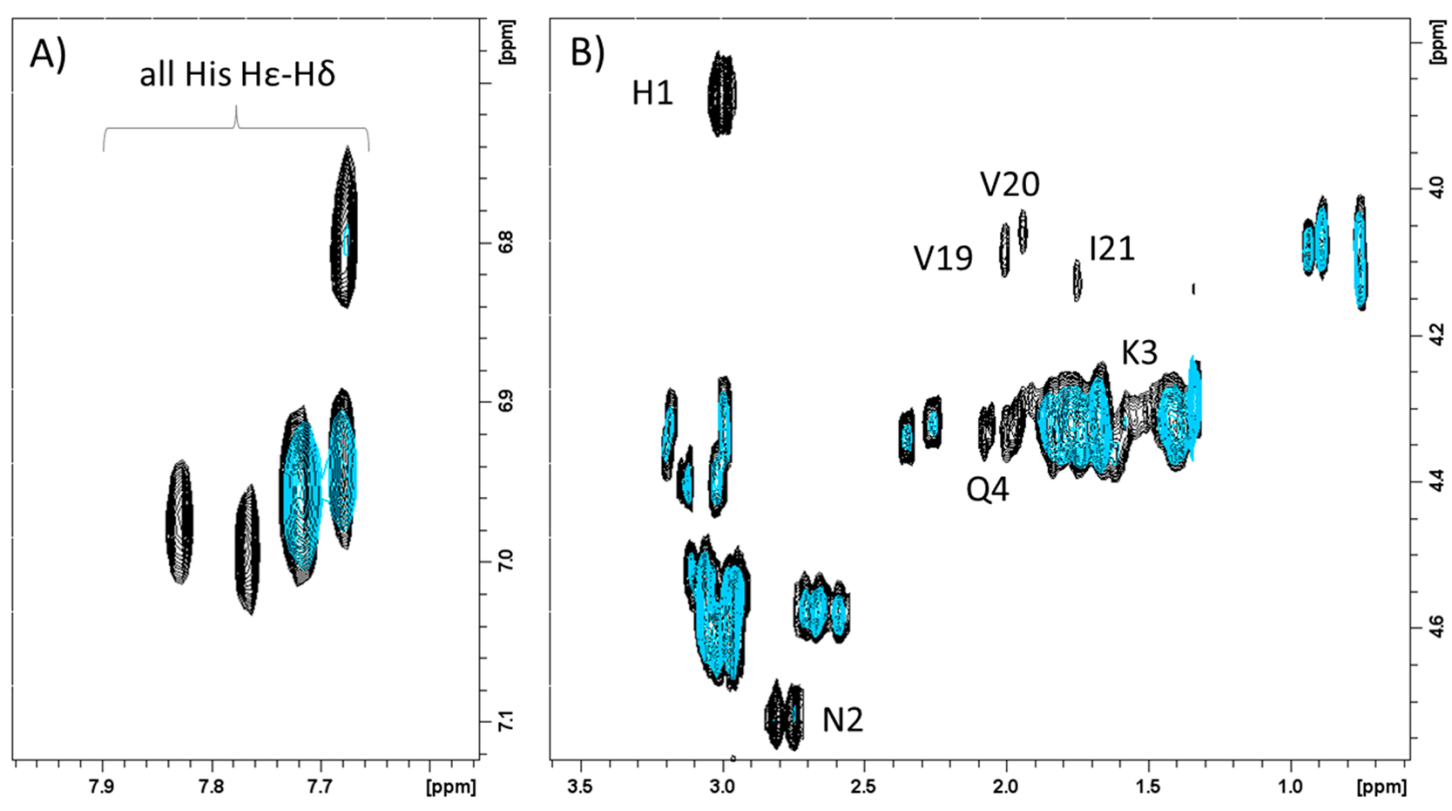
Superimposition of aromatic (A) and aliphatic (B) regions of ^1^H–^1^H TOCSY spectra of SgI-29, 1 mM in absence (black) and in the presence of 0.2 (light blue) equiv of Cu^2+^. *T* = 298 K, pH 7.4.

Loss of the next proton leads to CuH_10_L, where the third imidazole is engaged in coordination. Around pH 5, the prevailing species is CuH_9_L; at this point, the UV–vis d-d band shifts to 600 nm, suggesting that one more nitrogen is being involved in the coordination. Most probably, it belongs to the N-terminal amine group, resulting in an {3N_im_, NH_2_} type of coordination. Basing on spectroscopic data, up to pH 8, the geometry of the copper(II) complex does not change. On the other hand, the comparison of p*K*_*a*_ values of the ligand and corresponding complex form of the next species, CuH_8_L (6.24 and 5.46, respectively, see Table 1) implicates participation of another histidine residue in binding. Most likely, an equilibrium between (at least) two different forms exists in solution, involving different imidazoles in the {3N_im_, NH_2_} binding mode. In fact, in the aromatic region of the NMR ^1^H–^1^H TOCSY spectra taken at pH 7.4 (Figure 9A), only three out of five His H_δ_–H_ε_ correlations disappeared upon Cu^2+^ addition. In contrast, the other two cross-peaks are less affected and still visible, indicating that not all the seven His are engaged in copper interaction. Since Hα-Hβ signals of Phe-16 and Ala-28, together with Arg-18 cross-peaks, also remain unchanged after addition of copper(II), we may assume His-15, His-17, and His-29 as no coordinating imidazole (Figure 9B). Finally, the large broadening effects detected on His-1, Asn-2, and Gln-4 Hα-Hβ signals supports copper interactions with the N-terminal region of SgI-29 by means of histamine-like binding. All these findings support an analogue copper and zinc coordination mode as indicated by the structural model shown in Figure 6B.

Above pH 8, CuH_4_L, and with the increase of pH, CuH_3_L, species are formed, in which one and two amide nitrogens are involved in the coordination sphere. It is also confirmed by the appearance of characteristic d-d bands on the CD spectra with maxima of 550 nm at pH 8 and 9 (Figure 8B).^34^ The remaining loss of protons belongs to lysine side chain deprotonation, which has no impact on coordination.

#### 4.3 SgIIA (KQEGRDHDKSKGHFHMIVIHHKGGQAHHG)

Potentiometric measurements allowed us to determine 14 protonation constants for the SgIIA ligand. Starting from the most acidic ones, there are two aspartic acid residues with p*K*_*a*_ values of 2.77 and 3.00, glutamic acid with p*K*_*a*_ = 4.26, seven histidine residues with p*K*_*a*_ values of 5.2, 5.75, 5.96, 6.41, 6.59, 7.1, and 7.55 respectively, free N-terminus with p*K*_*a*_ = 9.47. Remaining constants belong to lysine residues with p*K*_*a*_ values of 10.21 and 10.34 (Table 1). As already seen for Sg-15 and SgI-29, all of the NMR spectra recorded for SgIIA indicate the absence of any specific structural rearrangement of the peptide in aqueous solution at both acidic and physiological pH.

#### 4.3.1 Zinc(II) Complexes

As in the previous cases, only mononuclear forms of Zn^2+^ complexes with SgIIA are present (ligand/metal ratio of 1:1; Supplementary Figure S7A,B).

**Figure 10:**
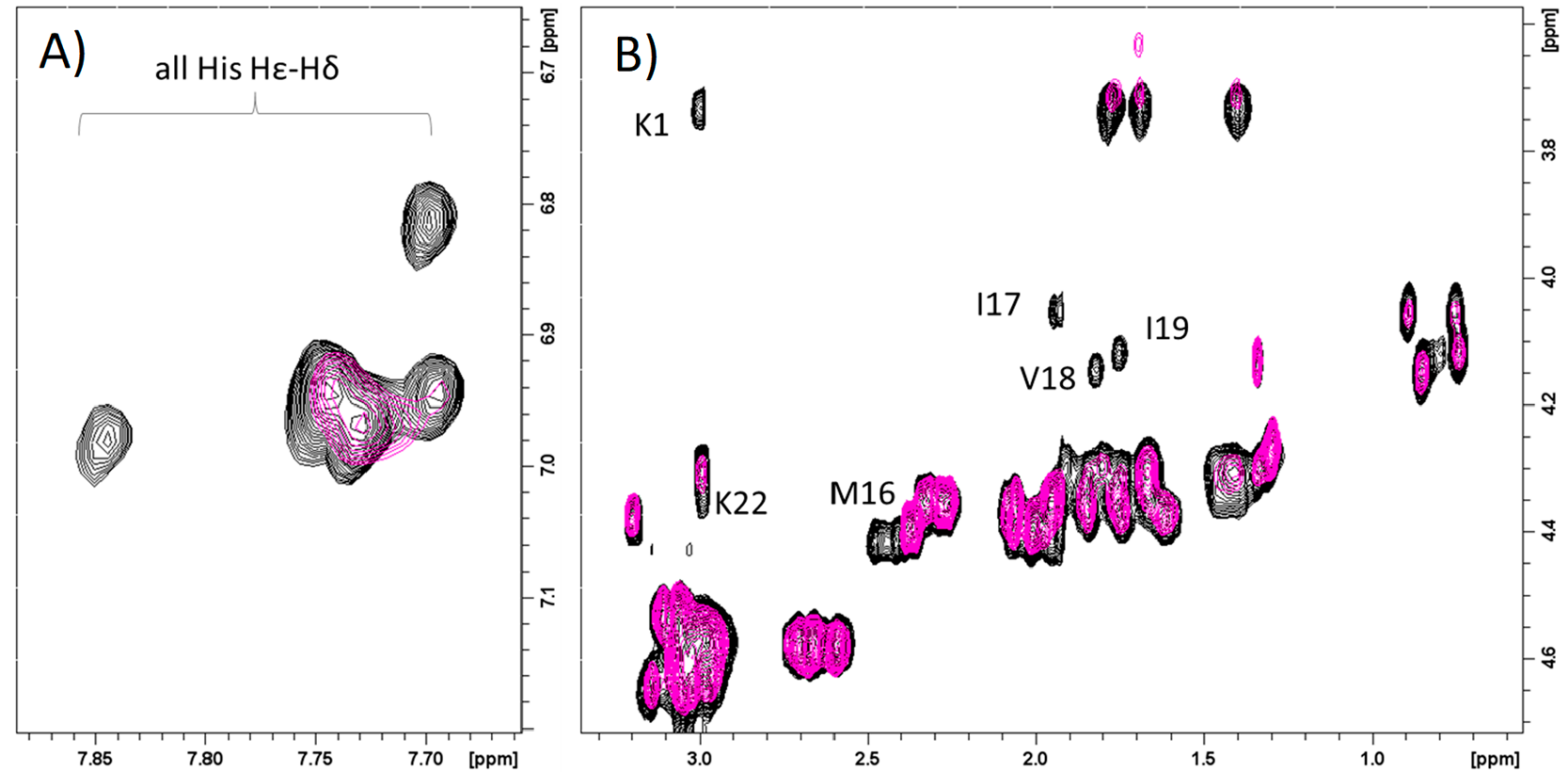
Superimposition of aromatic (A) and aliphatic (B) regions of ^1^H–^1^H TOCSY spectra of SgIIA, 1 mM in absence (black) and in the presence of 0.5 (magenta) equiv of Zn^2+^. *T* = 298 K, pH 7.4.

First observed complex form is ZnH_8_L with its maximum around pH 5.9 (Supplementary Figure S8A, Table 1). All acidic residues as well as the C-terminus are deprotonated at this point. Four out of seven histidine residues have also lost their protons either to simply deprotonation or involvement in zinc(II) binding. Up to the ZnH_4_L species, all the seven histidines and N-terminus are deprotonated and their p*K*_*a*_ values are lowered when compared to ligand itself. A closer look at the SgIIA sequence reveals that it comprises not two (like SgI-29), but three locations where histidines are accumulated close to each other: -**His**^13^-Phe^14^-**His**^15^-, -**His**^20^-**His**^21^, and -**His**^27^-**His**^28^. Since pointing out that the donor atom set was inconclusive using only potentiometry, we used NMR spectroscopy to determine the coordination mode at physiological pH, where ZnH_5_L species dominates in the solution. On the aromatic region (Figure 10A), where due to the overlap of signals we can identify five out of seven signals, we observe that at least two of them are less affected than others and most probably are not directly involved in coordination. Based on the aliphatic spectral region (Figure 10B), we can exclude His-7 and most probably also His-27 from being involved in Zn(II) binding since Asp-6 and Asp-8 signals as well as Ala-26 correlations remain unchanged. The most affected signals belong to the N-terminus Lys-1, Met-16, Ile-17, Val-18, Ile-19, and Lys-22. Such behavior supports that, at physiological pH, the most probable atom donor set for the zinc(II) complex belongs to the N-terminal amine nitrogen and three imidazoles of His-15, His-20, and His-21 (Figure 2C). It is also worth noting that an alternative set of imidazoles among all the seven His could be probable, and most likely, an equilibrium between different forms could take place.

With the increase of pH, three more deprotonations can be observed which result in the formation of ZnH_3_L, ZnH_2_L, and ZnHL species that are related to lysine side chain deprotonations and have no further impact on the complex coordination mode. Deprotonation of the most basic Lys constant was not detected in the calculations.

#### 4.3.2 Copper(II) Complexes

Similarly to the Zn^2+^-SgIIA complex, SgIIA forms only mononuclear complexes with Cu^2+^ (ligand/metal ratio of 1:1; Supplementary Figure S7C,D).

**Figure 11:**
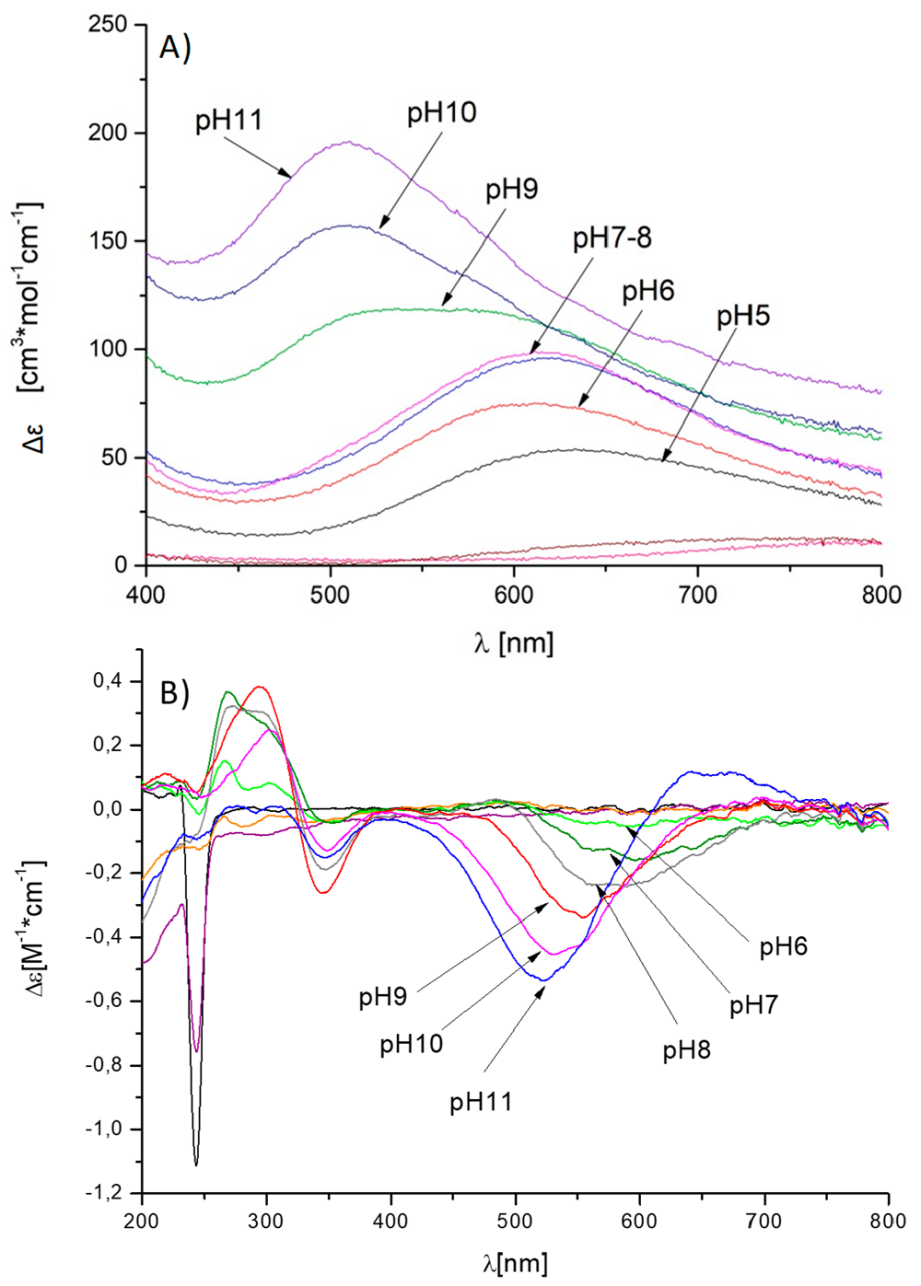
UV–vis (A) and CD (B) spectra of Cu^2+^ complexes with SgIIA in the range 200–800 nm. M/L ratio = 0.9:1.

The first complex observed during the potentiometric measurements is CuH_11_L (Supplementary Figure S8B, Table 1). In this form, the C-terminal group, aspartic and glutamic acid side chains and one imidazole are already deprotonated and do not take part in coordination. Loss of two protons leads to the formation of CuH_9_L, with a maximum at pH 4.8, in which two more imidazoles are deprotonated, and most probably, one of them is involved in coordination. The next species, which possesses a maximum at around pH 5.3, CuH_8_L, involves a second imidazole in binding. UV–vis spectroscopy confirms the 2N coordination near pH 5 with a band rising at 625 nm (Figure 11A).^27^ Around pH 6, the formation of the next three species can be observed: CuH_7_L with a maximum at pH 5.6, CuH_6_L with maximum at pH 6.1, and CuH_5_L with maximum at pH 6.6. The UV–vis band shifts to 610 nm, suggesting the involvement of another nitrogen in coordination sphere. At this point, the coordination mode most probably is a {3N_im_, NH_2_} set of donors. In SgIIA, again, the proximity of histidine residues in 3 locations in the sequence is observed. In order to establish the coordination mode at physiological pH, spectroscopic studies conducted. For the CuH_4_L species that dominate in solution above pH 7, the position of the mentioned UV–vis band does not change, yet it increases in intensity, and additionally, a d–d transition band is observed on the CD spectra (Figure 11B), suggesting amide binding and the initiation of a square planar geometry of the complex.^34^

**Figure 12:**
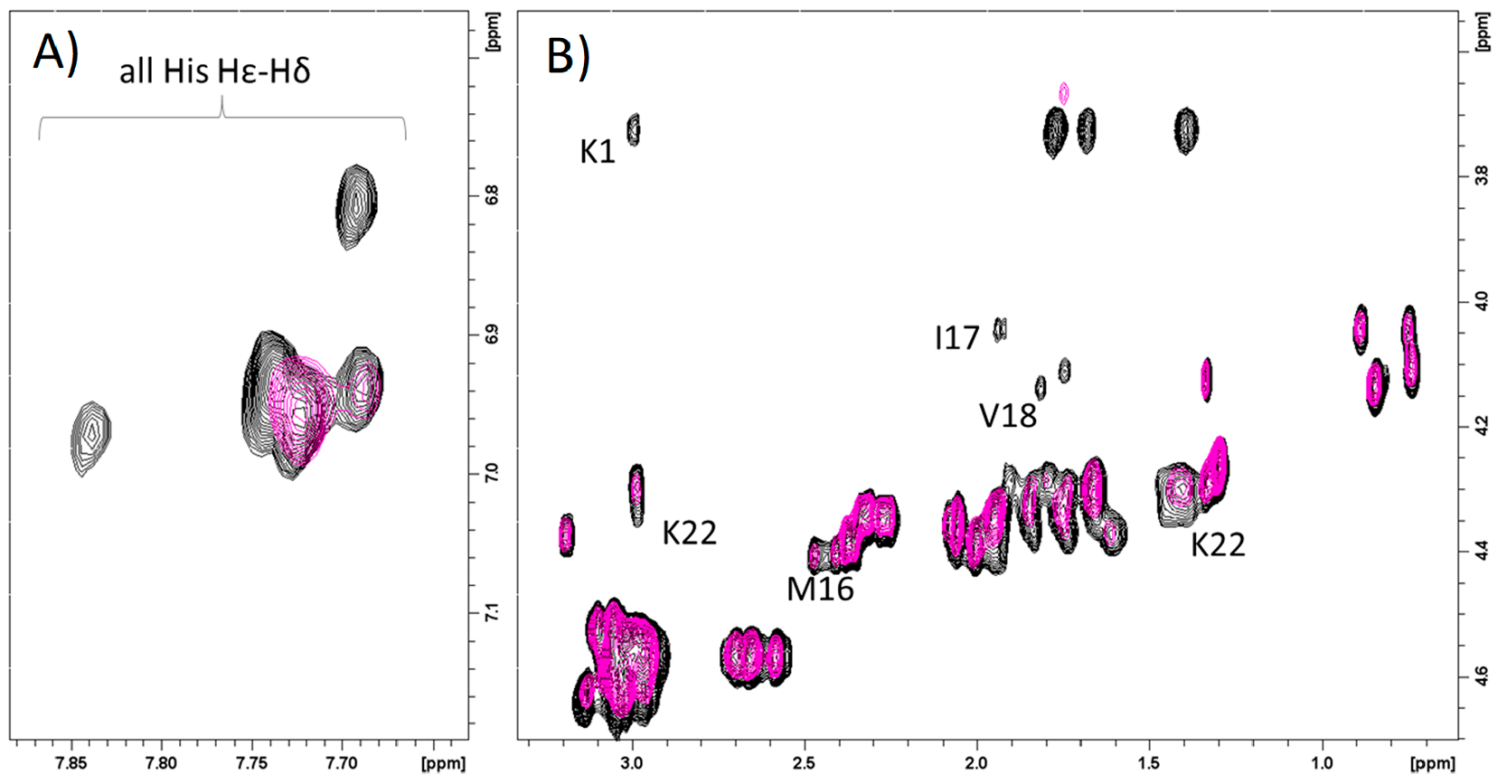
Superimposition of aromatic (A) and aliphatic (B) regions of ^1^H–^1^H TOCSY spectra of SgIIA, 1 mM in absence (black) and in the presence of 0.2 (magenta) equiv of Cu^2+^. *T* = 298 K, pH 7.4.

In the aromatic region of the NMR spectra acquired at pH 7.4 (Figure 12A), 3 out of seven histidine imidazoles are less affected than others. In the aliphatic region (Figure 12B), again, we do not observe a change in signals belonging to Gln-2, Glu-3, Arg-5, Asp-6, Asp-8, Gln-25, and Ala-26.

On the other hand, the most effected protons belong to the N-terminus, Lys-1, Met-16, Ile-17, Val-18, Ile-18, and Lys-22. All these data strongly suggest Lys-1 N-terminal amine nitrogen and His-15, His-20 and His-21 imidazole nitrogens as binding donor atoms (Figure 6C). With the increase of pH, amides enter the coordination sphere, and an equilibrium between {NH_2_, 3N_im_} and {N_im_, 2N_am_} coordination mode is possible. Basing on significant changes on Ile, Val, and Met signals, it can be concluded that the anchoring site is the imidazole from His-20 or His-21 and two preceding amide nitrogens. The last four deprotonations belong to lysine side chains and have no impact on copper(II) coordination.

#### 4.4 Antimicrobial Activity

*In vitro* antimicrobial activity of all studied semenogelins and their Cu^2+^ and Zn^2+^ complexes was tested against seven reference strains from the ATCC collection. In some cases, moderate activity against the *E. faecalis* strain was detected (with MIC50 = 256 μg/mL). Two facts are particularly interesting: (i) For the first time, antimicrobial activity of Cu^2+^-Sg-15 was detected. (ii) The biological activity of Sg-15 and SgIIA is induced by the binding of Cu^2+^ and/or Zn^2+^ (Table 2).

**Table 2:** Antimicrobial Activity (*In Vitro*) of Sg-15, SgI-29, and SgIIA and Their Cu^2+^ and Zn^2+^ Complexes against *E. faecalis* 29212, Assessed as MIC (μg/mL) Values at pH = 7.4^a^

	Sg-15	Sg-15-Cu^2+^	Sg-15-Zn^2+^	SgI-29	SgI-29-Cu^2+^	SgI-29-Zn^2+^	SgIIA	SgIIA-Cu^2+^	SgIIA-Zn^2+^
MIC50 (μg/mL) *E. faecalis* 29212	n/s	256	n/s	256	256	256	n/s	256	256

a Activity against other examined bacterial strains (*E. coli* 25922, *S. aureus* 43300, *P. aeruginosa* 27853, *K. pneumoniae* 700603, *A. baumannii* 19606), and *C. albicans* 10231 was not detected.

## 5 Discussion and Conclusions

**Figure 13:**
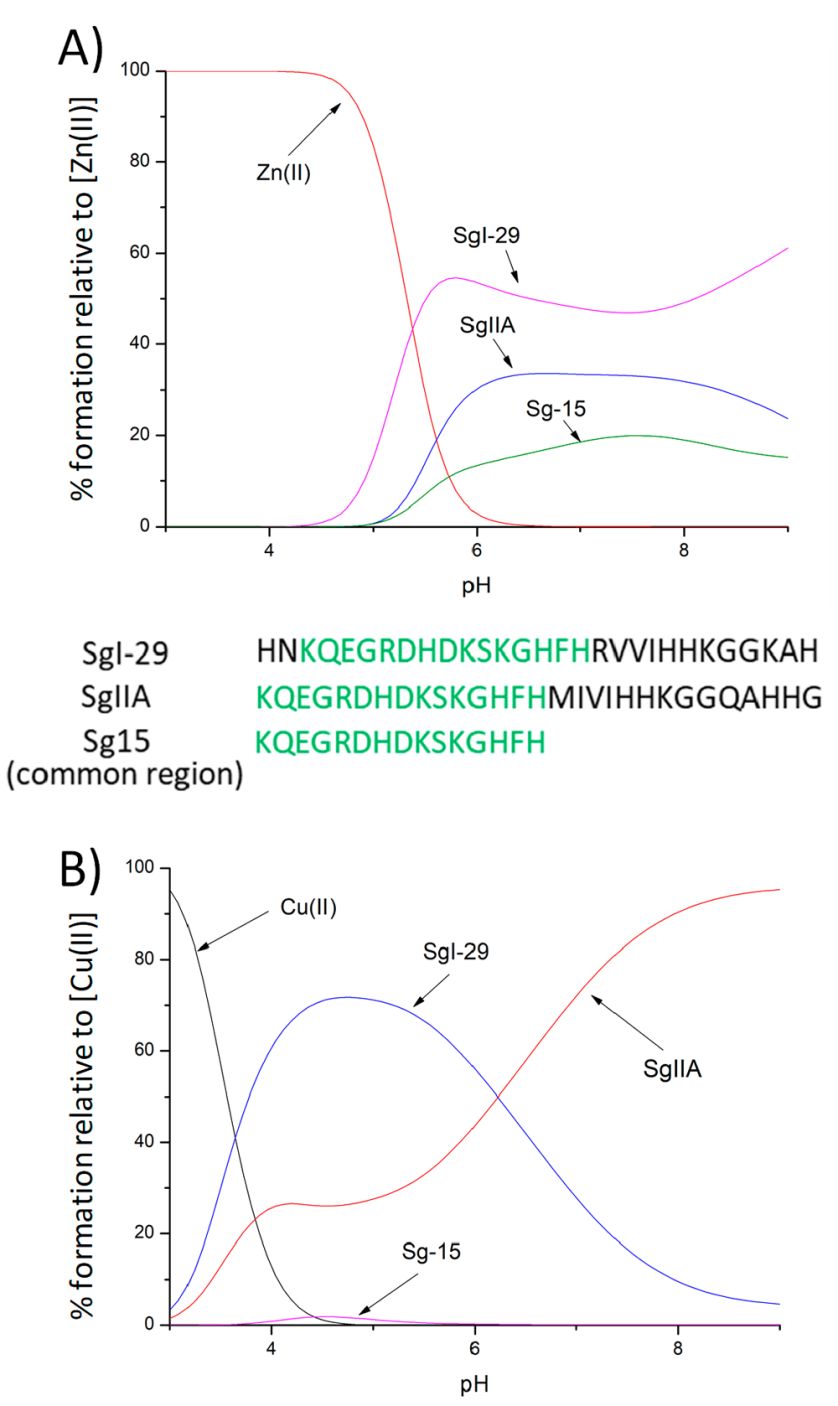
Competition diagrams for SgI-29, SgIIA, and Sg15 and Zn(II) (A) or Cu(II) (B), showing the relative amount of each complex at different pH values in a hypothetical situation, when equimolar amounts of reagents are mixed. Calculations are based on the binding constants from Table 1.

All the studied semenogelin fragments (SgI-29, SgIIA, and Sg-15) form stable complexes with Zn(II) and Cu(II) ions, in each case involving 3 histidine imidazoles and the N-terminal amino group in binding at physiological pH. For all three systems, zinc(II) and copper(II) coordination modes are very similar, supporting the anchoring role played by the N-terminus and His residues in metal binding. The stability of zinc(II) complexes is fairly comparable (Figure 13A), while in the case of copper(II), its histamine-like complex with SgI-29 strongly dominates in solution up to pH 6, above which the situation changes in favor of SgIIA, which, at basic pH, involves one His imidazole and three amides in complex formation (Figure 13B).

It is worth noting that in the case of SgIIA and Sg-15 (the common 15 AA fragment of SgI-29 and SgIIA), their antibacterial effect against *E. faecalis* is induced by the coordination of Cu^2+^ and/or Zn^2+^. For the first time, antimicrobial properties of the copper(II)-bound Sg-15 peptide were shown. The discussed MIC50 values (256 μg/mL) are not really of applicable potential, but they may suggest that metal binding is one of the ways in which nature regulates semenogelins’ antimicrobial potential.

**Figure 14:**
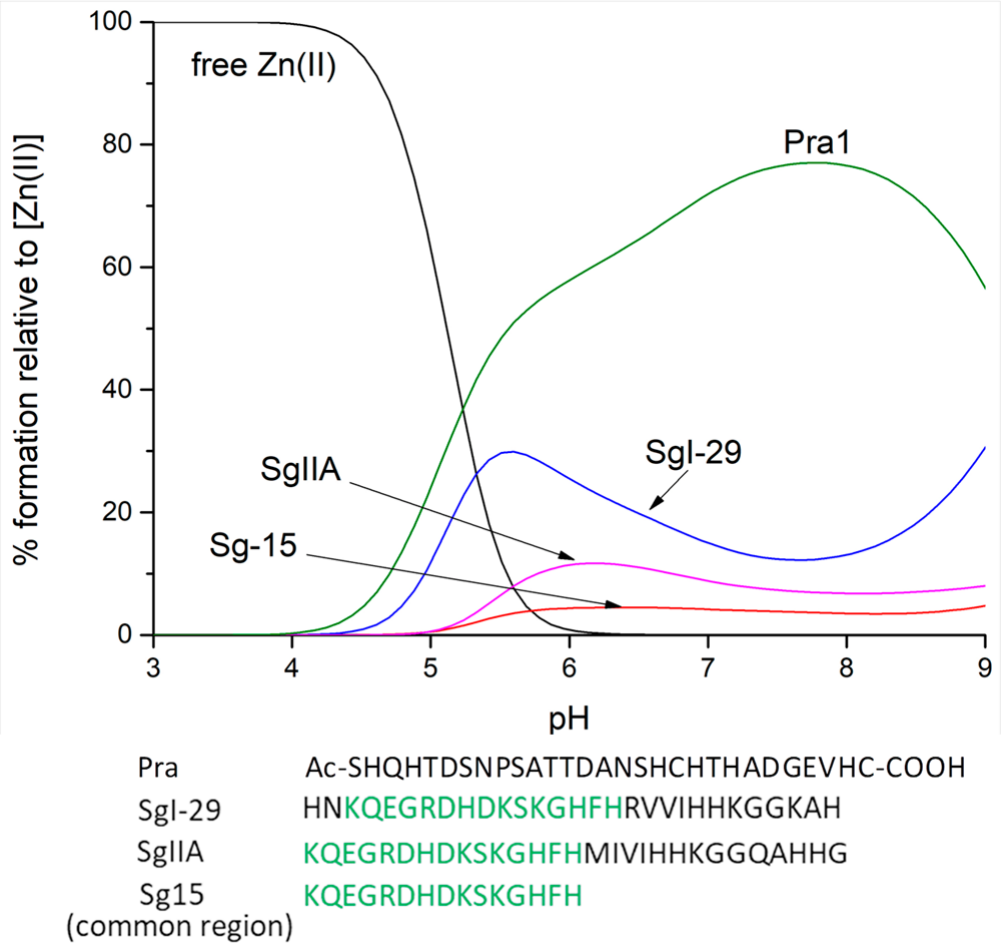
Competition diagrams for the C-terminal fragment of Pra1, SgI-29, SgIIA, Sg15, and Zn(II), showing the relative amount of each complex at different pH values in a hypothetical situation, when equimolar amounts of reagents are mixed. Calculations are based on the binding constants from Table 1 and ref (35).

Similar metal coordination modes of SgI-29 and SgIIA correlate quite well with their similar antimicrobial behavior, and therefore, their modes of action can be jointly discussed. Interestingly, the {NH_2_, 3N_im_} mode of Zn(II) coordination is thermodynamically less stable than the {4N_im_} one present in the Pra1 zincophore from *C. albicans* (Figure 14),^35^ thus suggesting that, since the metal–AMP complex stability cannot compete with that of the metallophore, the possible antimicrobial mode of action is most likely not based on nutritional immunity (scavenging of metal ions away from the pathogen).

Results of near-ultraviolet circular dichroism spectroscopy (data not shown) conclude that all ligands assume a disordered structure, regardless of their solution (even in SDS, which imitates the cell membrane environment and often induces the formation of an α-helix) and the presence of metal ions. Thus, the biological activity of semenogelins is not influenced by the adaptation of a typical secondary structure. There is also no correlation between the stability of the formed complexes and their antimicrobial activity; therefore, the mechanism of the antimicrobial action of semenogelins may not (solely) be explained by the process of nutritional immunity.

**Figure 15:**
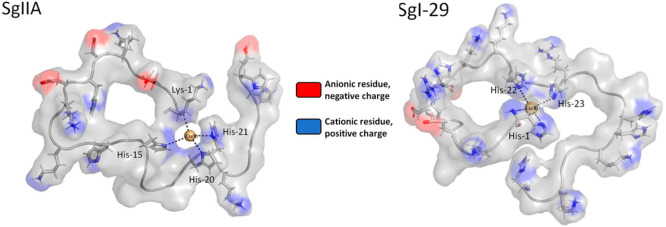
Diagrams of Cu^2+^-SgIIA (left) and Cu^2+^-SgI-29 (right) complexes showing their local charge distribution. Anionic fragments are marked in red; cationic fragments are marked in blue.

Quite likely, the antimicrobial mode of action is related to the cationic nature of these peptides, which facilitates interaction with the negatively charged bacterial membrane, and after reaching an appropriate concentration, it damages it, resulting in cell lysis. In the native SgI-29 and SgIIA semenogelins, the strong local positive charge in the metal-bound HH motif could contribute to the antimicrobial activity of the complexes. This hypothesis is an intriguing invitation to further studies on the mode of action of metal complexes of AMPs with the HH motif (Figure 15).

## Supporting Information Available

Comparison of SgI and SgII amino acid sequences, MS data, complex stability constants, distribution diagrams and additional spectroscopic data (PDF)

## Abbreviations

PSA: prostate-specific antigen;
TSB: tryptone soy broth;
CFU: colony-forming unit;
MIC: minimum inhibitory concentration;
TTC: 2,3,5-triphenyltetrazolium chloride;
MBC: minimum bactericidal concentration;
MFC: minimum fungicidal concentration
