# Supplementary material for: Semenogelins Armed in Zn(II) and Cu(II): May Bioinorganic Chemistry Help Nature to Cope with Enterococcus faecalis?
Source: Inorg Chem. 2023 Aug 15;62(34):14103–15. doi: 10.1021/acs.inorgchem.3c02390 (PMC10466376; doi:10.1021/acs.inorgchem.3c02390)
Supplement: Supplementary file 1 — ic3c02390_si_001.pdf [file ic3c02390_si_001.pdf]

Supporting Information

Semenogelins armed in Zn(II) and Cu(II) – may bioinorganic chemistry help nature to cope with *E. faecalis*?

*Dorota Dudek[1], Adriana Miller[1], Aleksandra Hecel[1], Arian Kola[2], Daniela Valensin[2], Aleksandra Mikołajczyk[3], Miquel Barcelo-Oliver[4], Agnieszka Matera-Witkiewicz[3], Magdalena Rowińska-Żyrek[1]**

[1] Faculty of Chemistry, University of Wrocław, 50-383 Wrocław, Poland

[2] Department of Biotechnology, Chemistry and Pharmacy, University of Siena, 53100 Siena, Italy

[3] Screening of Biological Activity Assays and Collection of Biological Material Laboratory, Wroclaw Medical University Biobank, Faculty of Pharmacy, Wrocław Medical University, 50-556 Wroclaw, Poland

[4] Department of Chemistry, University of Balearic Islands, 07122 Palma de Mallorca, Spain

```
SgI       1   MKPNIIFVLSLLLILEKQAAVMGQKGGSKGRLPSEFSQFPHGQKGQHYSGQKGKQQTESK    60
SgII      1   MKSIILFVLSLLLILEKQAAVMGQKGGSKGQLPSGSSQFPHGQKGQHYFGQKDQQHTKSK    60
SgI      61   GSFSIQYTYHVDANDHDQSRKSQQYDLNALHKTTKSQRHLGGSQQLLHNKQEGRDHDKSK   120
SgII     61   GSFSIQHTYHVDINDHDWTRKSQQYDLNALHKATKSKQHLGGSQQLLNYKQEGRDHDKSK   120
SgI     121   GHFHRVVIHHKGGKAHRGTQNPSQDQGNSPSGKGISSQYSNTEERLWVHGLSKEQTSVSG   180
SgII    121   GHFHMIVIHHKGGQAHHGTQNPSQDQGNSPSGKGLSSQCSNTEKRLWVHGLSKEQASASG   180
SgI     181   AQKGRKQGGSQSSYVLQTEELVANKQQRETKNSHQNKGHYQNVVEVREEHSSKVQTSLCP   240
SgII    181   AQKGRTQGGSQSSYVLQTEELVVNKQQRETKNSHQNKGHYQNVVDVREEHSSKLQTSLHP   240
SgI     241   AHQDKLQHGSKDIFSTQDELLVYNKNQHQTKNLNQDQQHGRKANKISYQSSSTEERRLHY   300
SgII    241   AHQDRLQHGPKDIFTTQDELLVYNKNQHQTKNLSQDQEHGRKAHKISYPSSRTEERQLHH   300
SgI     301   GENGVQKDVSQSSIYS--------------------------------------------   316
SgII    301   GEKSVQKDVSKGSISIQTEEKIHGKSQNQVTIHSQDQEHGHKENKISYQSSSTEERHLNC   360
SgI     316   ------------------------------------------------------------   316
SgII    361   GEKGIQKGVSKGSISIQTEEQIHGKSQNQVRIPSQAQEYGHKENKISYQSSSTEERRLNS   420
SgI     317   ----------------QTEEKAQGKSQKQITIPSQEQEHSQKANKISYQSSSTEERRLHY   360
SgII    421   GEKDVQKGVSKGSISIQTEEKIHGKSQNQVTIPSQDQEHGHKENKMSYQSSSTEERRLNY   480
SgI     361   GENGVQKDVSQRSIYSQTEKLVAGKSQIQAPNPKQEPWHGENAKGESGQSTNREQDLLSH   420
SgII    481   GGKSTQKDVSQSSISFQIEKLVEGKSQIQTPNPNQDQWSGQNAKGKSGQSADSKQDLLSH   540
SgI     421   EQKGRHQHGSHGGLDIVIIEQEDDSDRHLAQHLNNDRNPLFT                     462
SgII    541   EQKGRYKQESSESHNIVITEHEVAQDDHLTQQYNEDRNPIST                     582
```

Supplementary Figure S1. Comparison of SgI and SgII amino acid sequences, based on the UniProt database, with marked recurring motifs. The sequences overlap in 78%

A.

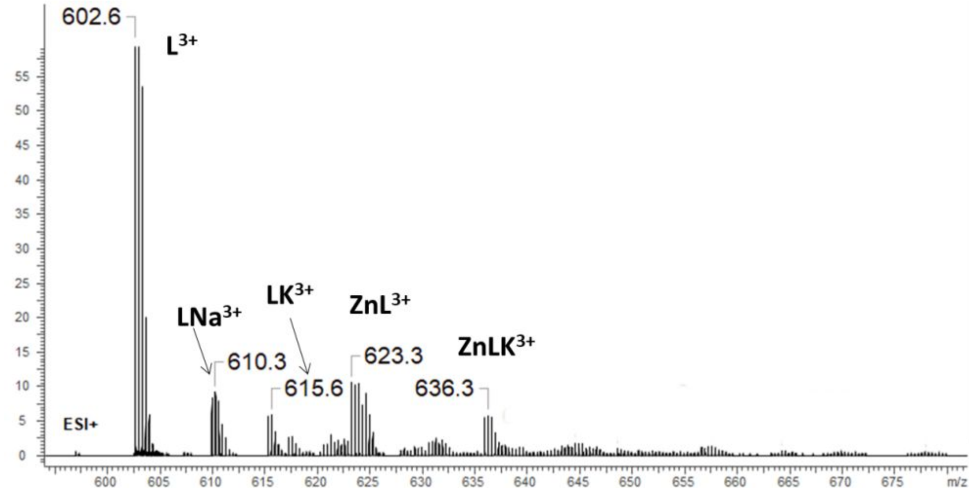
602.6
L3+
LNa3+
610.3
LK3+
615.6
ZnL3+
623.3
ZnLK3+
636.3
ESI+
m/z

B.

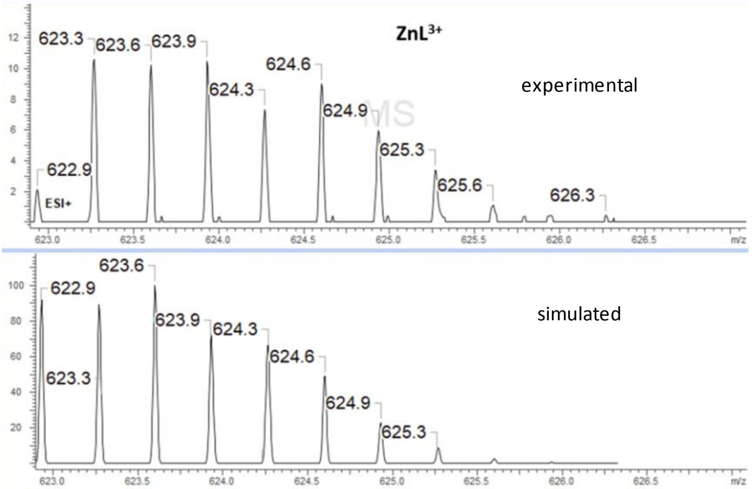
ZnL3+
experimental
simulated
ESI+
m/z

C.

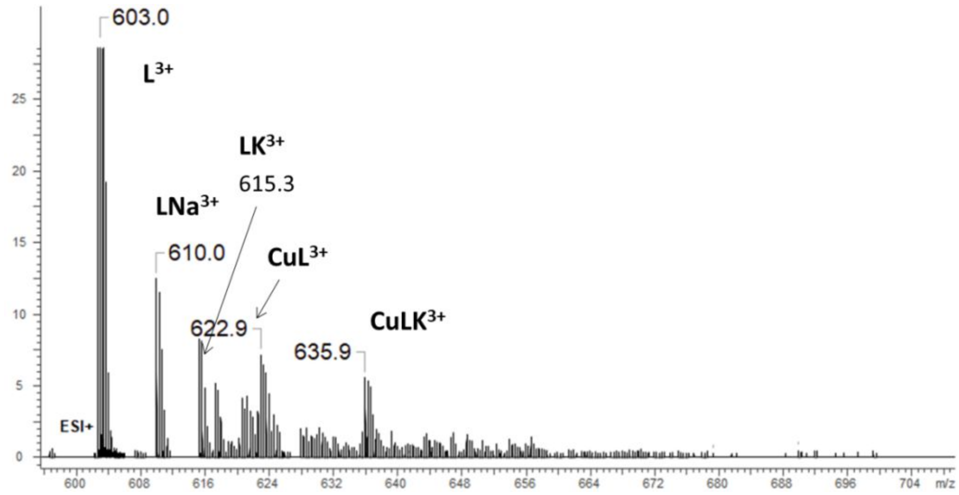

D.

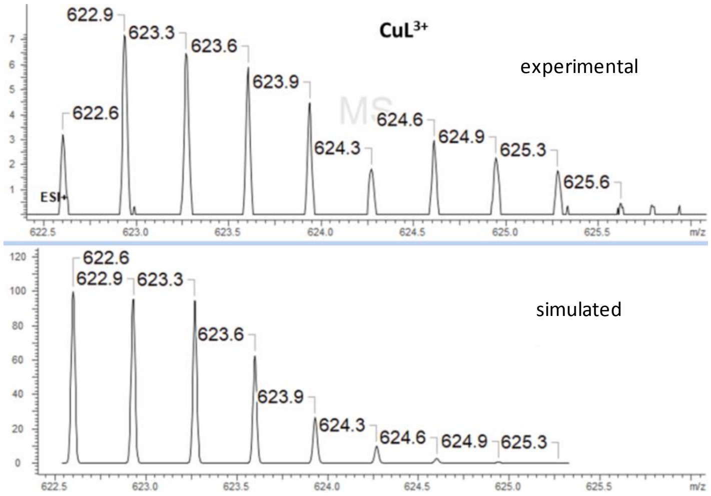

Supplementary Figure S2. MS spectra of Zn^{2+}-Sg-15 (A, B) and Cu^{2+}-Sg-15 (C, D) complexes. In the A spectrum (Zn^{2+}-Sg-15 complex), five main signals were assigned to: the single peptide ($m/z = 602.6$, $z = 3+$), its sodium ($m/z = 610.3$, $z = 3+$) and potassium adducts ($m/z = 615.6$, $z = 3+$), Zn^{2+}-Sg-15 complex ($m/z = 623.3$, $z = 3+$) and its potassium adduct ($m/z = 636.3$, $z = 3+$). In the C spectrum (Cu^{2+}-Sg-15 complex), signals from the ligand ($m/z = 603.0$, $z = 3+$), its sodium ($m/z = 610.0$, $z = 3+$) and potassium adduct ($m/z = 615.3$, $z = 3+$), Cu^{2+}-Sg-15 complex ($m/z = 622.9$, $z = 3+$) and its potassium adduct ($m/z = 635.9$, $z = 3+$) were observed.

In the B and D spectra, the experimental (top) and simulated (bottom) isotopic patterns are compared and clearly show the presence of the complex. Conditions: [Zn^{2+}] = [Cu^{2+}] = [Sg-15] = 3 x 10^{-4} M in a 1:1 methanol-water mixture; M^{2+}:peptide ratio was 1:1.

A.

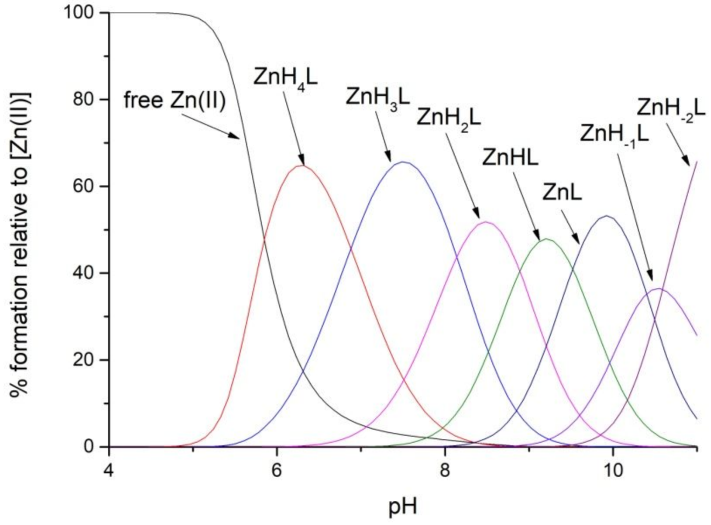

B.

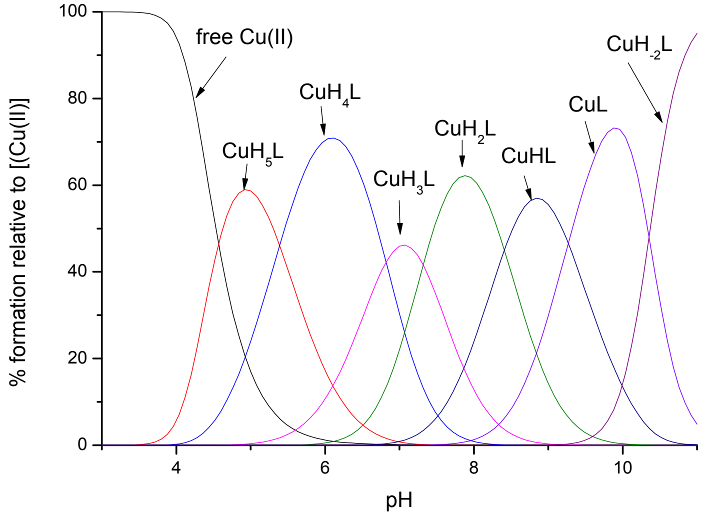

Supplementary Figure S3. Distribution diagram for the formation of A) Zn^{2+} and B) Cu^{2+} complexes with Sg-15 ligand at 25°C and $I = 0.1$ M. [L] = 0.5×10^{-3} M; M:L molar ratio of 0.9:1.

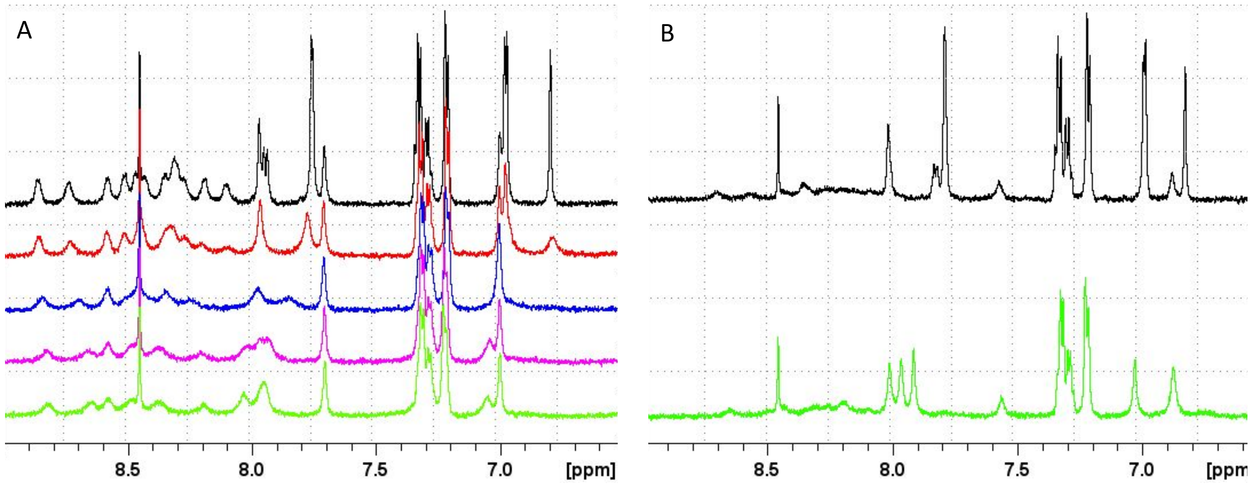

Figure S4. Superimposition of amide and aromatic regions of ^{1}H 1D spectra of Sg-15, 1 mM, pH 7.4 in absence (black) and in presence of 0.125 (red), 0.375 (blue), 0.625 (magenta) and 0.875 (green) Zn^{2+} eqs. (A) T= 278 K; (B) T=298 K.

A.

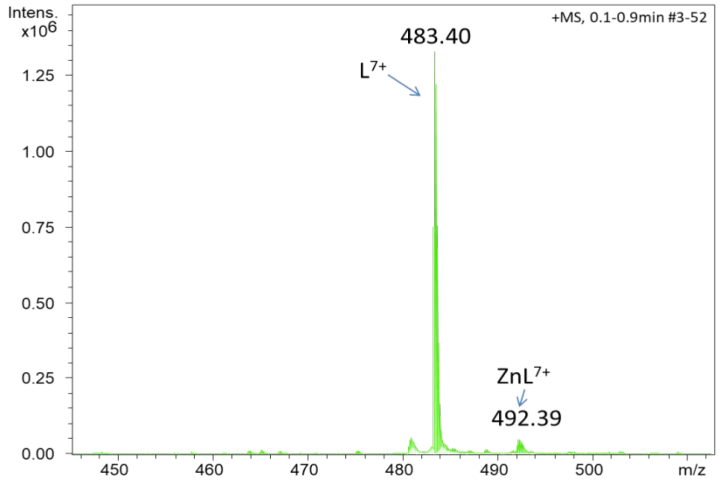
Intens.
x10^6
+MS, 0.1-0.9min #3-52
483.40
L7+
ZnL7+
492.39
1.25
1.00
0.75
0.50
0.25
0.00
450
460
470
480
490
500
m/z

B.

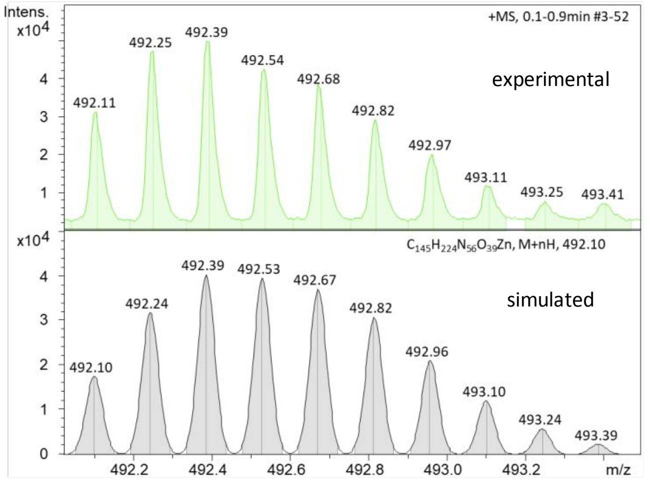
Intens.
x10^4
+MS, 0.1-0.9min #3-52
experimental
492.11
492.25
492.39
492.54
492.68
492.82
492.97
493.11
493.25
493.41
C145H224N56O39Zn, M+nH, 492.10
simulated
492.10
492.24
492.39
492.53
492.67
492.82
492.96
493.10
493.24
493.39
492.2
492.4
492.6
492.8
493.0
493.2
m/z

C.

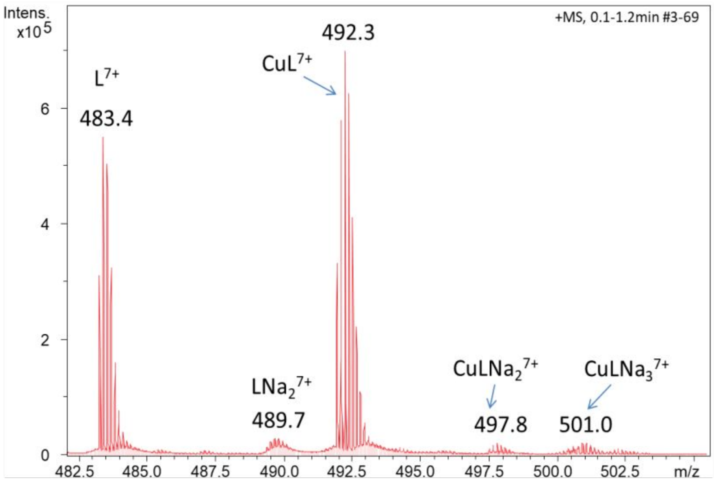

D.

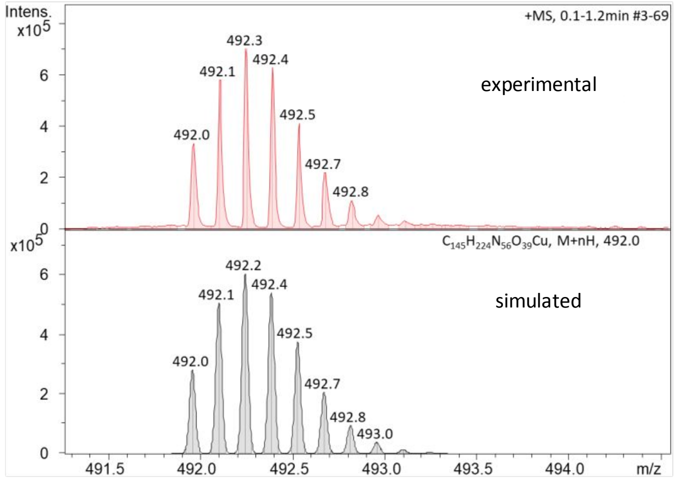

Supplementary Figure S5. MS spectra of Zn^{2+}-SgI-29 (A, B) and Cu^{2+}- SgI-29 (C, D) complexes. In the A spectrum (Zn^{2+}-SgI-29), only two signals were observed: for the ligand (*m/z* = 483.4, *z* = 7+) and its Zn^{2+} complex (*m/z* = 492.39, *z* = 7+). In the C spectrum (Cu^{2+}-SgI-29), five most intensive signals were assigned to the ligand (*m/z* = 483.4, *z* = 7+), Cu^{2+} complex (*m/z* = 492.3, *z* = 7+) and sodium adducts – both for ligand (*m/z* = 489.7, *z* = 3+), and complex (*m/z* = 497.8, *z* = 7+; *m/z* = 501.0, *z* = 7+). In the B and D spectra, the experimental (top) and simulated (bottom) isotopic patterns are compared and clearly show the presence of the complex. Conditions: [Zn^{2+}] = [Cu^{2+}] = [SgI-29] = 3 x 10^{-4} M in a 1:1 methanol-water mixture; M^{2+}:peptide ratio was 1:1

A.

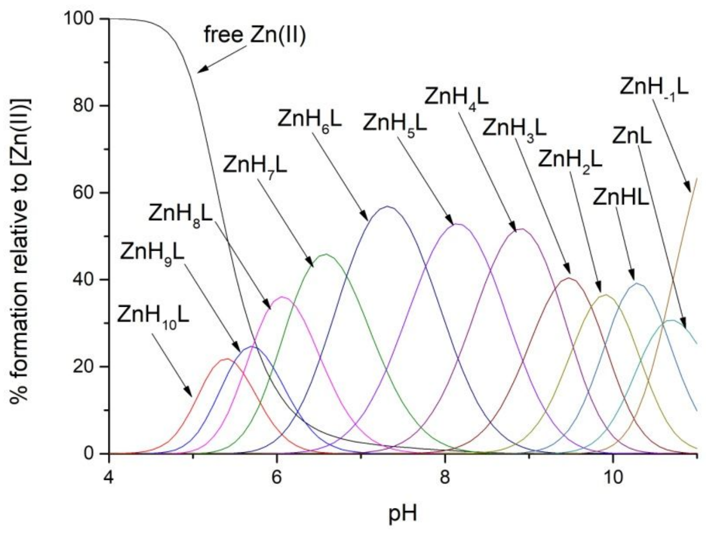

B.

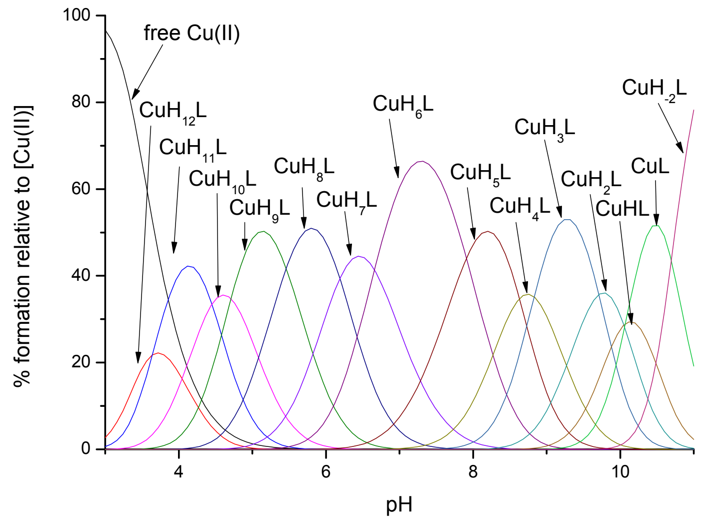

Supplementary Figure S6. Distribution diagram for the formation of A) Zn^{2+} and B) Cu^{2+} complexes with SgI-29 ligand at 25 °C and I = 0.1 M. [L] = 0.5 × 10^{-3} M; M:L molar ratio of 0.9:1.

A.

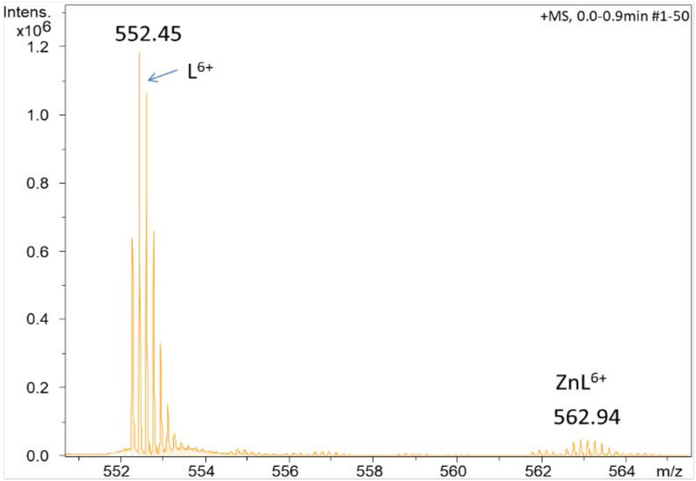
Intens.
x10^6
552.45
L^6+
ZnL^6+
562.94
+MS, 0.0-0.9min #1-50
m/z

B.

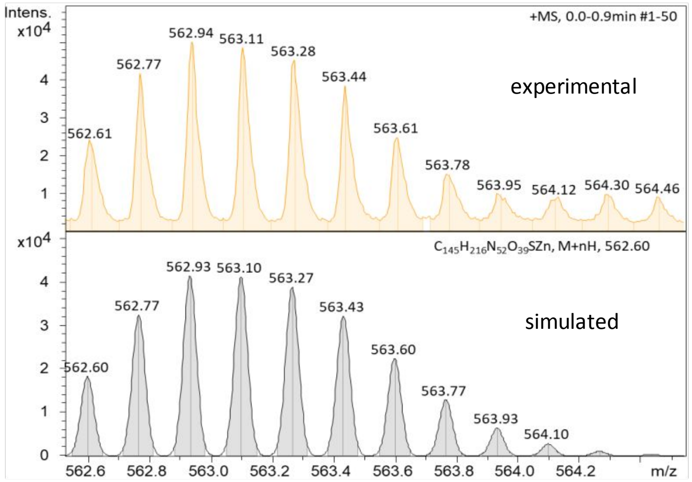
Intens.
x10^4
+MS, 0.0-0.9min #1-50
experimental
562.61
562.77
562.94
563.11
563.28
563.44
563.61
563.78
563.95
564.12
564.30
564.46
C145H216N52O39SZn, M+nH, 562.60
simulated
562.60
562.77
562.93
563.10
563.27
563.43
563.60
563.77
563.93
564.10
m/z

C.

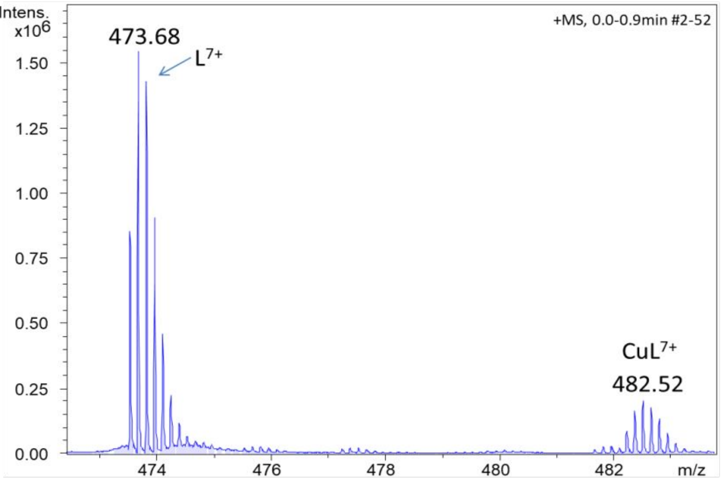

D.

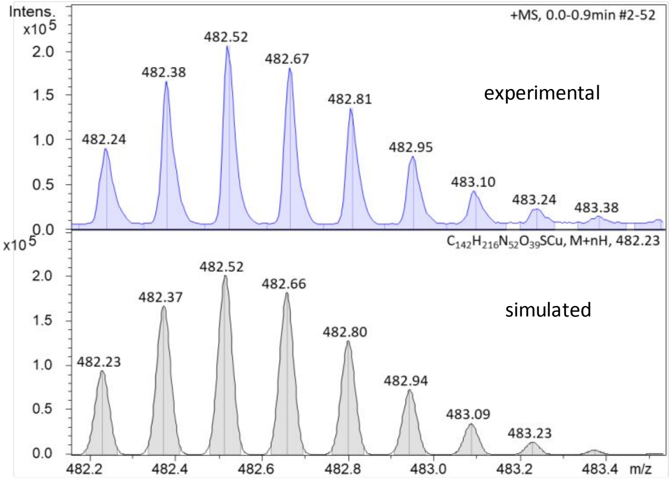

Supplementary Figure S7. MS spectra of Zn^{2+}-SgIIA (A, B) and Cu^{2+}-SgIIA (C, D) complexes. In the A spectrum (Zn^{2+}-SgIIA), signals from the ligand (m/z = 552.45, z = 6+) and its Zn^{2+} complex (m/z = 562.94, z = 6+) were observed. In the C spectrum (Cu^{2+}-SgIIA), also two main peaks were visible: for the ligand (m/z = 473.68, z = 3+) and Cu^{2+} complex (m/z = 482.52, z = 3+). In the B and D spectra, the experimental (top) and simulated (bottom) isotopic patterns are compared and clearly show the presence of the complex. Conditions: [Zn^{2+}] = [Cu^{2+}] = [SgIIA] = 3 x 10^{-4} M in a 1:1 methanol-water mixture; M^{2+}:peptide ratio was 1:1

A.

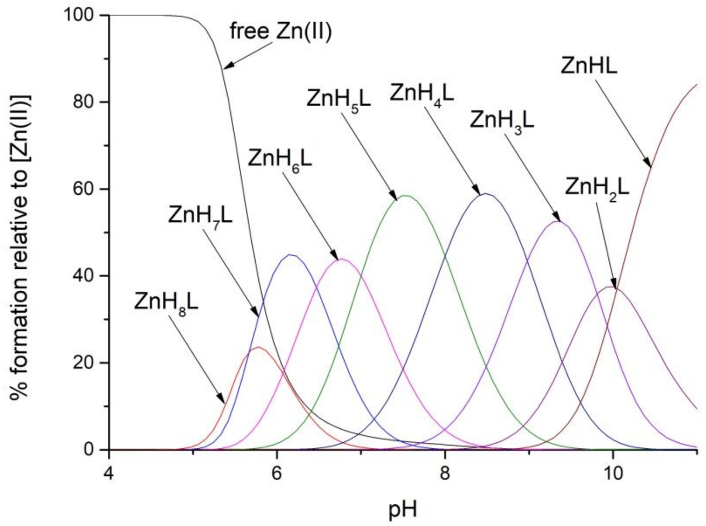

B.

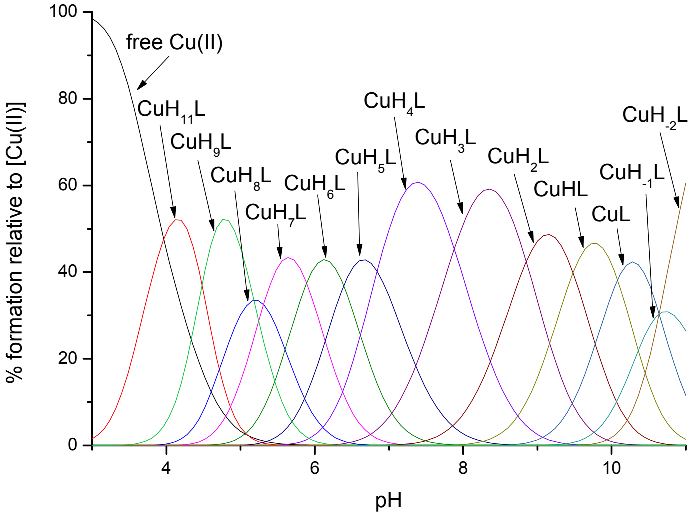

Supplementary Figure S7. Distribution diagram for the formation of A) Zn^{2+} and B) Cu^{2+} complexes with SgIIA ligand at 25°C and $I = 0.1$ M. [L] = 0.5×10^{-3} M; M:L molar ratio of 0.9:1.

Supplementary Table S1 Literature data indicating concentrations of metals in semen samples.

minimal value	maximal value	mean	reference
Zn(II) ions			
72 mg/l (1.10 mM)	187 mg/l (2.860 mM)		1
20.95 mg/l (0.32 mM)	423 mg/l (6.47 mM)	96.80 mg/l (1.48 mM)	2
Cu(II) ions			
0.99 mg/l (0.016 mM)	23.12 mg/l (0.364 mM)	7.54 mg/l (0.119 mM)	2
0.183 mg/l (0.003 mM)	0.194 mg/l (0.003 mM)		3

Supplementary Table S2. Potentiometric and spectroscopic data for Cu(II) and Zn(II) complexes with ligand Sg-15, SgI-29 and SgIIA. $T = 298$ K, $I = 0.1$ mol dm^{-3} ($NaClO_4$) and M : L molar ratio = 0.9:1. Values in parentheses are standard deviations on the last significant figure

Species	logβ	pK_a	UV-Vis		CD	
			λ [nm]	ε [M^{-1}cm^{-1}]	λ [nm]	Δε [M^{-1}cm^{-1}]
Sg-15						
LH	10,66 (3)	10,66				
LH_2	21,19 (1)	10,53				

**LH_3**	30,85 (2)	9,66				
**LH_4**	38,43 (2)	7,58				
**LH_5**	45,53 (2)	7,1				
**LH_6**	51,99 (2)	6,46				
**LH_7**	58,05 (2)	6,06				
**LH_8**	62,41 (2)	4,36				
**LH_9**	65,99 (2)	3,58				
**LH_{10}**	69,15 (3)	3,16				
**LH_{11}**	71,49 (4)	2,34				
**zinc(II) complexes**						
**ZnH_4L**	44,08 (1)					
**ZnH_3L**	37,20 (2)	6,88				
**ZnH_2L**	29,06 (3)	8,14				
**ZnHL**	20,17 (3)	8,89				
**ZnL**	10,65 (3)	9,52				
**$ZnH_{-1}L$**	0,25 (4)	10,4				
**$ZnH_{-2}L$**	-10,34 (3)	10,59				
**copper(II) complexes**						
**CuH_5L**	52,48 (1)		641	47,04	minor	
**CuH_4L**	47,09 (1)	5,39	631	59,44	minor	
**CuH_3L**	40,27 (2)	6,82	612	65,25	minor	
**CuH_2L**	32,95 (2)	7,32	578	67,39	562	-0,25
					338	-0,21
					246	1,53
**CuHL**	24,54 (3)	8,41	521	82,64	544	-0,64
					336	-0,27
					248	2,57
**CuL**	15,27 (2)	9,27	511	96,60	530	-1,00
					340	-0,09
					246	2,71
**$CuH_{-2}L$**	-5,44 (2)		510	112,70	528	-1,15
					302	0,27
					248	2,81
**SgI-29**						
**LH_2**	22,24 (4)					
**LH_3**	32,71 (2)	10,47				
**LH_4**	43,09 (3)	10,38				
**LH_5**	52,98 (2)	9,89				

**LH_6**	62,43 (3)	9,45				
**LH_7**	69,82 (3)	7,39				
**LH_8**	76,75 (3)	6,93				
**LH_9**	83,24 (3)	6,49				
**LH_{10}**	89,48 (3)	6,24				
**LH_{11}**	95,30 (3)	5,82				
**LH_{12}**	100,82 (3)	5,52				
**LH_{13}**	105,71 (3)	4,89				
**LH_{14}**	109,45 (3)	3,74				
**LH_{15}**	112,67 (3)	3,22				
**LH_{16}**	114,87 (3)	2,20				
**LH_{17}**	117,05 (4)	2,18				
**zinc(II) complexes**						
**$ZnH_{10}L$**	93,34 (3)					
**ZnH_9L**	87,85 (3)	5,49				
**ZnH_8L**	82,14 (2)	5,71				
**ZnH_7L**	75,94 (1)	6,2				
**ZnH_6L**	69,11 (1)	6,83				
**ZnH_5L**	61,34 (1)	7,77				
**ZnH_4L**	52,80 (1)	8,54				
**ZnH_3L**	43,49 (2)	9,31				
**ZnH_2L**	33,75 (3)	9,74				
**ZnHL**	23,68 (4)	10,07				
**ZnL**	13,09 (5)	10,59				
**$ZnH_{-1}L$**	2,49 (3)	10,6				
**copper(II) complexes**						
**$CuH_{12}L$**	105,41 (3)		minor	minor	676	0,15
					336	-0,18
					241	-1,14
**$CuH_{11}L$**	101,77 (1)	3,64	634	42	313	-0,08
**$CuH_{10}L$**	97,32 (2)	4,45				
**CuH_9L**	92,61 (1)	4,71	599	105,78	658	0,11
					312	-0,15
**CuH_8L**	87,15 (1)	5,46	599	107,26	673	0,16
					168	-0,19
**CuH_7L**	80,96 (1)	6,19				
**CuH_6L**	74,3 (1)	6,66	698	116,45	672	0,15
					338	-0,18

**CuH_5L**	66,39 (2)	7,91	582	127,15	554	-0,07
					491	0,13
					341	-0,47
					285	0,58
**CuH_4L**	57,76 (3)	8,63				
**CuH_3L**	48,93 (2)	8,83	535	155,86	549	-0,26
					482	0,12
					343	-0,63
					293	0,59
**CuH_2L**	39,22 (3)	9,71				
**CuHL**	29,17 (3)	10,05	524	200,12	541	-0,36
					344	-0,47
					298	0,44
**CuL**	19,1 (2)	10,07				
**$CuH_{-2}L$**	-2,29 (1)		524	205,18	532	-0,50
					347	-0,15
					304	0,35
					274	0,37
**SgIIA**						
**LH_2**	22,84 (4)					
**LH_3**	33,18 (3)	10,34				
**LH_4**	43,39 (4)	10,21				
**LH_5**	52,86 (3)	9,47				
**LH_6**	60,41 (3)	7,55				
**LH_7**	67,51 (4)	7,1				
**LH_8**	74,10 (3)	6,59				
**LH_9**	80,51 (4)	6,41				
**LH_{10}**	86,47 (3)	5,96				
**LH_{11}**	92,22 (3)	5,75				
**LH_{12}**	97,42 (3)	5,2				
**LH_{13}**	101,68 (3)	4,26				
**LH_{14}**	104,68 (3)	3				
**LH_{15}**	107,45 (3)	2,77				
**zinc(II) complexes**						
**ZnH_8L**	78,58 (2)					
**ZnH_7L**	72,91 (1)	5,67				
**ZnH_6L**	66,44 (1)	6,47				
**ZnH_5L**	59,44 (1)	7				

**ZnH_4L**	51,44 (1)	8				
**ZnH_3L**	42,46 (2)	8,98				
**ZnH_2L**	32,65 (2)	9,81				
**ZnHL**	22,60 (1)	10,05				
**ZnL**						
**copper(II) complexes**						
**$CuH_{11}L$**	97,29 (1)		minor		minor	
**$CuH_{10}L$**			minor		minor	
**CuH_9L**	88,33 (1)		632	107,09	minor	
**CuH_8L**	83,15 (1)	5,18				
**CuH_7L**	77,84 (1)	5,31				
**CuH_6L**	71,95 (1)	5,89	618	148,97	599	-0,06
					303	0,08
					267	0,15
**CuH_5L**	65,56 (1)	6,39				
**CuH_4L**	58,72 (1)	6,84	614	191,53	594	-0,16
					352	-0,04
					297	0,27
					269	0,37
**CuH_3L**	50,84 (1)	7,88	614	196,23	566	-0,24
					348	-0,19
					297	0,30
					271	0,32
**CuH_2L**	41,99 (1)	8,85	548	237,41	554	-0,34
					345	-0,26
					296	0,38
**CuHL**	32,51 (1)	9,48				
**CuL**	22,43 (1)	10,08	509	374.45	532	-0,45
					348	-0,13
					304	0,24
**$CuH_{-1}L$**	11,79 (1)	10,64				
**$CuH_{-2}L$**	1,15 (1)	10,64	509	451,38	522	-0,54
					348	-0,15

References

1. M. B. Sørensen, I. A. Bergdahl, N. H. Hjøllund, J. P. Bonde, M. Stoltenberg, E. Ernst, "Zinc, magnesium and calcium in human seminal fluid: relations to other semen parameters and fertility." Molecular human reproduction 5.4 (1999): 331-337.

2. I. Bolanca, J. Obhodas, D. Ljiljak, L. Matjacic, K. Kuna, "Synergetic effects of K, Ca, Cu and Zn in human semen in relation to parameters indicative of spontaneous hyperactivation of spermatozoa." PLoS One 11.3 (2016): e0152445.

3. F. Jockenhövel, M. Bals-Pratsch, H. P. Bertram, E. Nieschlag, "Seminal lead and copper in fertile and infertile men." Andrologia 22.6 (1990): 503-511.
